# Conditional antagonism in co-cultures of *Pseudomonas aeruginosa* and *Candida albicans*: An intersection of ethanol and phosphate signaling distilled from dual-seq transcriptomics

**DOI:** 10.1371/journal.pgen.1008783

**Published:** 2020-08-19

**Authors:** Georgia Doing, Katja Koeppen, Patricia Occipinti, Colleen E. Harty, Deborah A. Hogan

**Affiliations:** Geisel School of Medicine at Dartmouth, Hanover, New Hampshire, United States of America; The University of Texas Health Science Center at Houston, UNITED STATES

## Abstract

*Pseudomonas aeruginosa* and *Candida albicans* are opportunistic pathogens whose interactions involve the secreted products ethanol and phenazines. Here, we describe the role of ethanol in mixed-species co-cultures by dual-seq analyses. *P*. *aeruginosa* and *C*. *albicans* transcriptomes were assessed after growth in mono-culture or co-culture with either ethanol-producing *C*. *albicans* or a *C*. *albicans* mutant lacking the primary ethanol dehydrogenase, Adh1. Analysis of the RNA-Seq data using KEGG pathway enrichment and eADAGE methods revealed several *P*. *aeruginosa* responses to *C*. *albicans*-produced ethanol including the induction of a non-canonical low-phosphate response regulated by PhoB. *C*. *albicans* wild type, but not *C*. *albicans adh1*Δ/Δ, induces *P*. *aeruginosa* production of 5-methyl-phenazine-1-carboxylic acid (5-MPCA), which forms a red derivative within fungal cells and exhibits antifungal activity. Here, we show that *C*. *albicans adh1*Δ/Δ no longer activates *P*. *aeruginosa* PhoB and PhoB-regulated phosphatase activity, that exogenous ethanol complements this defect, and that ethanol is sufficient to activate PhoB in single-species *P*. *aeruginosa* cultures at permissive phosphate levels. The intersection of ethanol and phosphate in co-culture is inversely reflected in *C*. *albicans*; *C*. *albicans adh1*Δ/Δ had increased expression of genes regulated by Pho4, the *C*. *albicans* transcription factor that responds to low phosphate, and Pho4-dependent phosphatase activity. Together, these results show that *C*. *albicans*-produced ethanol stimulates *P*. *aeruginosa* PhoB activity and 5-MPCA-mediated antagonism, and that both responses are dependent on local phosphate concentrations. Further, our data suggest that phosphate scavenging by one species improves phosphate access for the other, thus highlighting the complex dynamics at play in microbial communities.

## Author summary

*Pseudomonas aeruginosa* and *Candida albicans* are opportunistic pathogens that are frequently isolated from co-infections. Using a combination of dual-seq transcriptomics and genetics approaches, we found that ethanol produced by *C*. *albicans* stimulates the PhoB regulon in *P*. *aeruginosa* asynchronously with activation of the Pho4 regulon in *C*. *albicans*. We validated our result by showing that PhoB plays multiple roles in co-culture including orchestrating the competition for phosphate and the production of 5-methyl-phenazine-1-carboxylic acid; the *P*. *aeruginosa* phenazine response to *C*. *albicans*-produced ethanol depends on phosphate availability. The conditional stimulation of antifungal production in response to sub-inhibitory concentrations of ethanol only under phosphate limitation highlights the importance of considering nutrient concentrations in the analysis of co-culture interactions and suggests that the low-phosphate response in one species influences phosphate availability for the other.

## Introduction

*Pseudomonas aeruginosa* and *Candida albicans* are opportunistic pathogens that are frequently isolated from co-infections [1–11]. These pathogens affect each other’s behaviors by competing for nutrients [12–17], making physical contact [3, 4, 7, 13, 14], secreting diffusible signaling molecules [18–23] and producing antimicrobials [18, 21, 24–30]. Studies highlighting the dynamic interplay between *P*. *aeruginosa* and *C*. *albicans* have contributed to the growing understanding of how interactions between microbes influence their physiology and behavior as well as microbiological and pathological outcomes.

Like many fermentative organisms, *C*. *albicans* produces ethanol. Ethanol is a biologically-active metabolite which, at sub-inhibitory concentrations, modulates *P*. *aeruginosa* behavior in multiple ways: it induces activity of the sigma factor AlgU through DksA and ppGpp signaling [31]; it promotes Pel matrix production through the Wsp system [29]; it decreases flagellar-mediated motility through a surface-sensing pathway [29, 32]; it affects programs known to contribute to *P*. *aeruginosa* virulence [29, 33]; and it fuels fungal antagonism [29]. The broad effects of ethanol apply to many contexts and the response of *P*. *aeruginosa* to *C*. *albicans-*produced ethanol can serve as a model for how *P*. *aeruginosa* may respond to other fermentative fungi and bacteria. We seek to further understand this response and identify common themes which may be implicated in other microbial interactions.

We used *P*. *aeruginosa* anti-fungal phenazine production to study the effects of ethanol in co-culture. Previous work has shown that ethanol stimulates the production and secretion of the phenazine 5-methyl-phenazine-carboxylic acid (5-MPCA) by *P*. *aeruginosa* and that, in return, *P*. *aeruginosa* phenazines promotes *C*. *albicans* fermentative metabolism and ethanol production [24–26]. *P*. *aeruginosa* does not normally secrete 5-MPCA in axenic cultures but in co-culture it does so through the MexGHI-OmpD efflux complex. Consequently, 5-MPCA enters *C*. *albicans* cells wherein it reacts with the basic amines such as arginine, disrupting protein function and forming a red pigment whose accumulation causes redox stress and eventually death of *C*. *albicans* [24, 27, 34]. While it is known that ethanol production by the fungus is necessary for *P*. *aeruginosa* 5-MPCA release, the mechanisms by which 5-MPCA production is regulated have not yet been described. The mechanisms of stimulation and ensuing consequences of *P*. *aeruginosa* 5-MPCA production and accumulation of the red 5-MPCA-derivative within *C*. *albicans* cells is a scopic case study for microbial interactions because it is an indicator of general antagonism.

Several studies have described the conditional production of antagonistic factors in response to nutrient availability [35–52]. This is often mediated transcriptionally, and such is the case for the low-phosphate response which is mediated by the PhoR-PhoB two-component system. Briefly, inorganic phosphate is sensed through the periplasmic domain of the phosphate transport complex, PstS, that is dependent on the ATPases PstA and PstB. The failure to bind phosphate in low-phosphate environments causes the de-repression of the sensor kinase PhoR which phosphorylates the response regulator PhoB and initiates PhoB DNA-binding to the promoters of many genes, including those in the phenazine biosynthetic pathway, and consequent transcriptional activation. The *P*. *aeruginosa* low-phosphate response includes the secretion of an arsenal of phosphatases, phospholipases and DNases that cleave phosphate from diverse macromolecules [40].

In the context of microbial communities, the secretion of these enzymes renders phosphate freely available to any nearby organism. Simultaneous production of antagonistic factors could aid *P*. *aeruginosa* in the competition for phosphate amongst other microbes. Indeed, in response to low phosphate and other complex stimuli, *P*. *aeruginosa* produces antagonistic factors like phenazines and phospholipases which play important roles in microbial interactions [39, 53, 54]. How neighboring microbes may alter each other’s low-phosphate responses is an open question but it has been reported that *P*. *aeruginosa* tailors its low-phosphate response to secondary stimuli: in *P*. *aeruginosa*, PhoB interacts with other regulators such as the transcription factor TctD [55] and the sigma factor VreI [37] to orchestrate the expression of its target genes. However, the mechanism by which PhoB exerts condition-specific control over its diverse regulon to manage antagonistic factors in microbial interactions is not yet fully understood.

Analysis of co-culture transcriptomic data from complex environments using curated pathways can be challenging if the complex conditions are not well represented by the data used for pathway definition. Furthermore, pathway definition relies on expert-contributed annotations, yet ~38% (2,162 of 5,704) of genes for the PAO1 reference strain (pseudomonas.com) lack descriptions. Recent methods have used unsupervised machine learning to leverage large amounts of transcriptomic data and automatically identify sets of genes with correlated expression patterns across large compendia of samples, agnostic of gene annotations and previously characterized pathways [53, 56–64]. In particular, the data-driven tool eADAGE has identified transcriptional signals that contain uncharacterized genes, manifest as small magnitude changes in expression, or are condition-specific.

Here, we demonstrate that *P*. *aeruginosa* and *C*. *albicans* undergo transcriptional changes in response to one another that are dependent on *C*. *albicans* ethanol production. Using eADAGE analysis, we identified a group of PhoB-regulated genes as differentially expressed in response to ethanol, and validated the result using genetic and biochemical assays. Both ethanol and PhoB activity were necessary for the production of the *P*. *aeruginosa* antifungal 5-MPCA, and ethanol was sufficient to stimulate PhoB in *P*. *aeruginosa* mono-cultures. Further, by examining *C*. *albicans* gene expression profiles from the same co-cultures, we found that the *C*. *albicans* low-phosphate response is inversely correlated to that of *P*. *aeruginosa*. In summary, we show that *P*. *aeruginosa* only produces antifungal 5-MPCA when *C*. *albicans* is not secreting extracellular phosphatases and the death of neighboring fungi would simultaneously remove a competitor and provide an increase in the availability of the essential nutrient phosphate. We conclude that the enmity of *P*. *aeruginosa*–*C*. *albicans* interactions is conditional upon phosphate concentrations, and further, that an induction of a phosphate limitation response in one species may improve access to phosphate in the other.

## Results

### Ethanol is a defining factor in *P*. *aeruginosa*—*C*. *albicans* interactions, promoting phenazine-mediated antagonism

**Fig 1:**
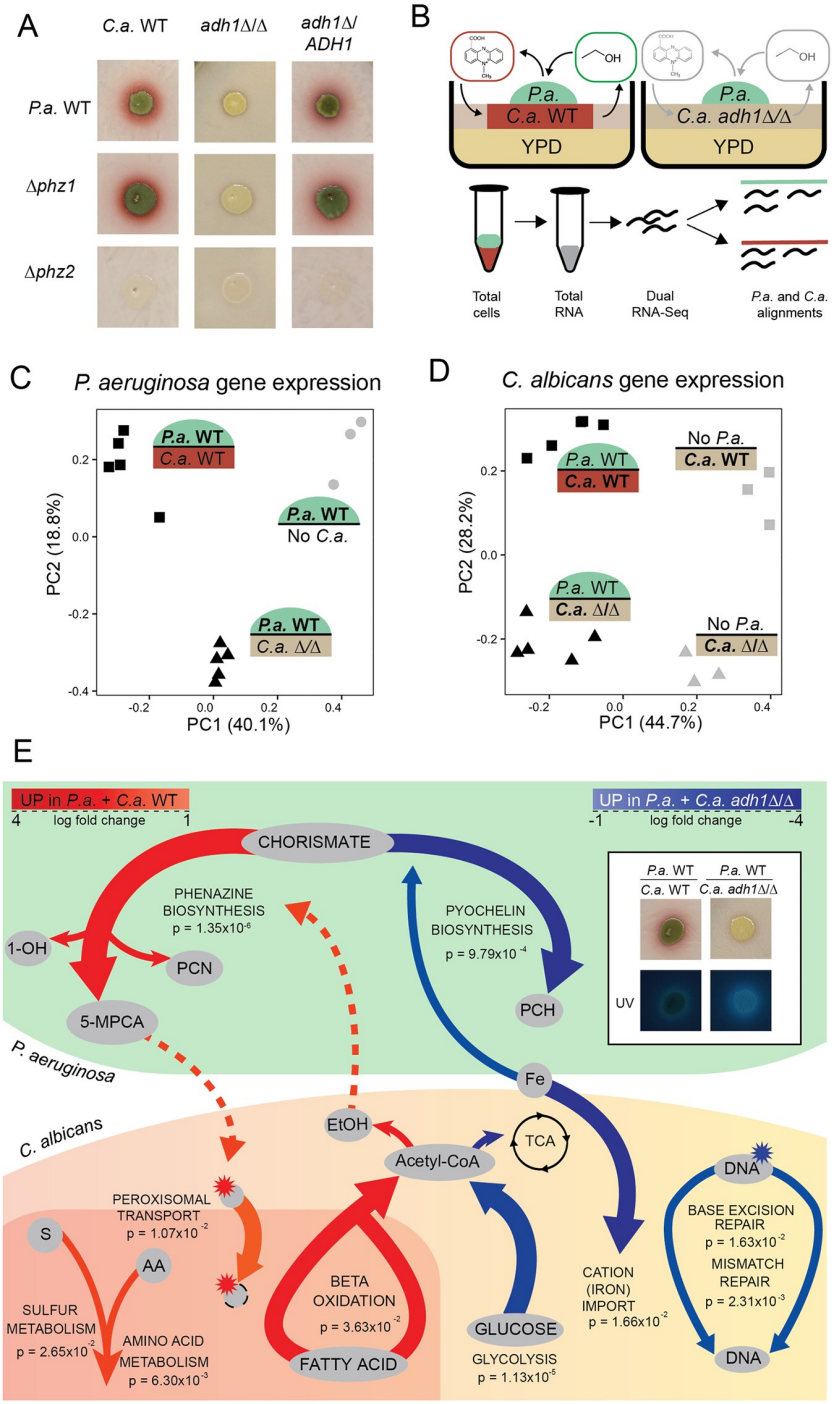
In co-culture of *C*. *albicans* (*C*.*a*.) and *P*. *aeruginosa* (*P*.*a*.), *C*.*a*.-produced ethanol stimulates *P*.*a*. to produce 5-MPCA and transcriptional responses ensue from both organisms. A) Co-cultures of *P*.*a*. wild type (WT) and mutants lacking phenazine biosynthesis operons (Δ*phz1* or Δ*phz2*) were inoculated onto 72 h-old lawns of *C*.*a*. WT, *adh1*Δ/Δ and *adh1*Δ/Δ reconstituted with *ADH1* (*adh1*Δ/*ADH1*). The red pigmentation indicates production of the phenazine 5-MPCA by *P*.*a*. B) Dual-seq allowed for parallel analyses of *P*.*a*. (green) and *C*.*a*. (red or pink) mRNA expression profiles from co-culture lawns to survey the effects of ethanol (green oval) and 5-MPCA (red oval) on gene expression. C) Principle component analysis (PCA) of TPM (transcripts per kilobase per million reads) from transcriptome profiles of *P*.*a*. grown alone (No *C*.*a*.), *P*.*a*. grown with *C*.*a*. WT, and *P*.*a*. grown with *C*.*a*. *adh1*Δ/Δ. D) PCA of gene expression profiles of *C*.*a*. WT and *C*.*a*. *adh1*Δ/Δ grown in mono-culture (No *P*.*a*.) or co-cultures with *P*.*a*. WT. E) Pathway analysis of DEGs from co-cultures of *P*.*a*. with *C*.*a*. WT compared to with *C*.*a*. *adh1*Δ/Δ. *P*.*a*. pathways were phenazine (PCN, 1-OH-P, 5-MCPA) biosynthesis and pyochelin (PCH) biosynthesis pathways. Inset shows increase in siderophore-derived fluorescence of co-cultures of *P*.*a*. with *C*.*a*. *adh1*Δ/Δ which is consistent with increased PCH production. *C*.*a*. pathways included amino acid metabolism, sulfur (S) metabolism, peroxisomal transport, fatty acid beta-oxidation, glycolysis, cation (e.g. Fe) import, base excision and mismatch DNA repair. Red arrows indicate higher expression in co-cultures with *C*.*a*. WT and blue arrows indicate higher expression in co-cultures with *C*.*a*. *adh1*Δ/Δ. P-values are from hypergeometric over-representation tests, FDR corrected.

When grown on a lawn of *C*. *albicans*, *P*. *aeruginosa* produces the anti-fungal phenazine 5-MPCA which is taken up by *C*. *albicans* and modified within the fungal cells to form a red derivative [24, 27, 29] that can be seen first below and then as a halo surrounding *P*. *aeruginosa* colonies (Fig 1A). The 5-MPCA precursor phenazine-1-carboxylic acid (PCA) can be synthesized via enzymes encoded in either of the two highly similar operons, *phzA1B1C1D1E1F1G1* (*phz1*) and *phzA2B2C2D2E2F2G2* (*phz2*) with different regulation; *phz1* contributes to phenazine production in liquid while *phz2* is responsible for phenazine production in colony biofilms [65]. Analysis of phenazine production in co-culture colony biofilms found that *phz1* was dispensable for the formation of red pigment, while *phz2* was required (Fig 1A). Consistent with previous results, 5-MPCA-derived red pigment formation required *phzM* [27], *mexGHI-ompD* and *soxR* [24], and was over-abundant upon inhibition of the conversion of 5-MPCA into another phenazine vis deletion of *phzS* [66] (S1A Fig). We have previously reported, and reproduced here, that *P*. *aeruginosa* 5-MPCA production required *C*. *albicans* ethanol production as *C*. *albicans adh1*Δ/Δ, which lacks the major ethanol dehydrogenase, did not elicit 5-MPCA-derived red pigment accumulation [29]. Chromosomal complementation of a single copy of *ADH1* in *C*. *albicans* restored 5-MPCA-derived red pigment production (Fig 1A). Consistent with previous reports [26, 67], HPLC analysis of *C*. *albicans* culture supernatants showed ethanol accumulation, and here we found that deletion of *ADH1* led to a 10-fold decrease in ethanol concentrations (S2A Fig). There was not a significant difference in concentrations of acetate or glycerol, two other *C*. *albicans* fermentation products when WT was compared to *adh1*Δ/Δ (S2B Fig).

To determine how *C*. *albicans* Adh1 activity influenced *P*. *aeruginosa* and how 5-MPCA-derived red pigment accumulation influenced *C*. *albicans*, we took a dual-seq approach: we collected total RNA for simultaneous transcriptome-wide analyses of both organisms from 16 h co-culture colony biofilms of *P*. *aeruginosa* with *C*. *albicans* WT, in which 5-MPCA-derivatives accumulated, and *P*. *aeruginosa* with *C*. *albicans adh1*Δ/Δ, in which 5-MPCA products were not observed (see Fig 1B for experimental set up). Single-species *P*. *aeruginosa* and *C*. *albicans* colony biofilms grown on YPD medium were also analyzed at the same time point. Principle component analysis of gene expression for each organism differentiated mono-culture and co-culture with the first component PC1, which accounted for 40.1% and 44.7% of total variance for *P*. *aeruginosa* and *C*. *albicans* respectively (Fig 1C and 1D). The presence of *ADH1* in *C*. *albicans* constituted a defining feature of co-culture captured by PC2 for both organisms, which accounting for 18.8% and 28.2% of total variance for *P*. *aeruginosa* and *C*. *albicans* respectively (Fig 1C and 1D).

### How fungal ethanol shapes co-culture transcriptomes: The *C*. *albicans* perspective

*C*. *albicans* Adh1 is responsible for reducing acetaldehyde to ethanol during fermentation, and we thus expected metabolic shifts between *C*. *albicans* WT and *adh1*Δ/Δ [29]. We identified differentially expressed genes (DEGs) between co-cultures of *C*. *albicans* WT and *adh1*Δ/Δ with *P*. *aeruginosa* (S1 Dataset) and conducted KEGG [68–70] pathway over-representation analysis. The *C*. *albicans* KEGG pathway for fatty acid beta-oxidation was over-represented in the DEGs and the genes it contained (e.g. *FAA2-1*, *FAA2-3*) were more highly expressed in *C*. *albicans* WT than in *C*. *albicans adh1*Δ/Δ (Fig 1E for pathway view, S2 Dataset for over-representation analysis). These results are consistent with a previous metabolomics study that reported a phenazine and other mitochondrial inhibitors increasing beta-oxidation in *C*. *albicans* WT [71].

Other KEGG pathways over-represented in DEGs between *C*. *albicans* WT and *adh1*Δ/Δ from co-cultures with *P*. *aeruginosa* were amino acid metabolism (e.g. *PUT2*, *GLT1*), sulfur metabolism (e.g. *MET15*) and peroxisomal transport (e.g. *PEX1*, *PEX19*) (Fig 1E, S2 Dataset). These pathways converge on reactive oxygen species (ROS) mitigation, genes of which trended toward upregulation (e.g. *GSH1*, *CAT1*). Since previous reports have shown that phenazines cause redox stress to neighboring fungi [25, 26], the upregulation of these pathways could have been due to ethanol-induced *P*. *aeruginosa* 5-MPCA production.

The KEGG pathway for glycolysis (e.g. *HXK2*, *PGI1*, *TDH3*, *PGK1*) was also over-represented in DEGs between *C*. *albicans* WT and *adh1*Δ/Δ from co-cultures with *P*. *aeruginosa* with the genes more highly expressed in *C*. *albicans adh1*Δ/Δ. This pathway was also more highly expressed in *C*. *albicans adh1*Δ/Δ grown alone, likely as metabolic compensation for the inability to ferment to ethanol (Fig 1E, S2 Dataset). There was also over-representation of the KEGG pathways for iron scavenging (e.g. *FRP1*, *FET99*) and the DNA damage response genes (e.g. *RBT5*, *CSA1*), with these genes more highly expressed in *C*. *albicans adh1*Δ/Δ (Fig 1E, S2 Dataset). Since these pathways were over-represented in DEGs between *C*. *albicans* WT and *adh1*Δ/Δ in both co- and mono-culture, they could represent direct or indirect effects of altered *C*. *albicans* metabolism or *P*. *aeruginosa* secreted products, inclusively: *C*. *albicans* intracellular metabolic products at high concentrations in *adh1*Δ/Δ, such as methylglyoxal [72, 73], and phenazines both cause oxidative stress leading to DNA damage.

### How fungal ethanol shapes co-culture transcriptomes: The *P*. *aeruginosa* perspective

We identified *P*. *aeruginosa* DEGs when grown on *C*. *albicans* WT compared to on *adh1*Δ/Δ. On *C*. *albicans* WT, *P*. *aeruginosa* upregulated genes involved in 5-MPCA biosynthesis including *phzM*, which is consistent with differences in 5-MPCA-derived red pigment formation between the two co-cultures (Fig 1E, S3 Dataset). While the last four genes of the *phz* operons have fewer than three SNPs between each gene pair and are thus not differentiated by alignment, *phzA1*, *phzB1 and phzC1* have substantial enough differences in sequence from *phzA2*, *phzB2 and phzC2* respectively that the two operons can be distinguished, and we found that transcripts from both *phz1* and *phz2* operons were more highly abundant by at least 2-fold when *P*. *aeruginosa* was grown in co-culture on *C*. *albicans* WT relative to on *adh1*Δ/Δ. Although *phzS* and *phzH* are not required for 5-MPCA biosynthesis [24, 29], they have been reported to have coordinated expression with other phenazine genes [55, 74] (S1D Fig for pathway) and indeed we saw increases in their expression on *C*. *albicans* WT compared to on *adh1*Δ/Δ as well (Fig 1E, S2 Dataset).

We again conducted KEGG pathway over-representation analysis and found over-representation of three KEGG pathways in DEGs from *P*. *aeruginosa* grown in co-culture with *C*. *albicans* WT compared to with *C*. *albicans adh1*Δ/Δ: phenazine biosynthesis, quorum sensing (QS), and pyochelin biosynthesis (Fig 1E, S2 Dataset). Over-representation of the phenazine biosynthesis pathway was expected based on the upregulation of the *phz* genes as described above. The identification of QS as an over-represented pathway was not surprising in light of the known regulation of phenazine biosynthesis by QS in response to environmental cues [75, 76], including in *C*. *albicans* co-culture [27]. *P*. *aeruginosa* QS involves three major transcriptional regulators: LasR [77], PqsR [78] and RhlR [77]. RhlR and PqsR, along with the PQS biosynthesis enzyme PqsA, were necessary for phenazine production (S1B Fig). Although Δ*lasR* appeared to produce less 5-MPCA than *P*. *aeruginosa* WT on *C*. *albicans* WT, consistent with previous data, it produced an abundance of the blue-green phenazines such as pyocyanin and PCN (S1D Fig) [18]. Upon examining the expression of gene targets for these transcription factors (S4 Dataset for gene lists and sources), we did not find evidence for a uniform change in QS that accompanied the activation of phenazine biosynthesis genes (S1C Fig).

The third over-represented KEGG pathway was the biosynthesis of pyochelin, a siderophore [51]. Expressly, genes involved in pyochelin biosynthesis, import and regulation were lower in *P*. *aeruginosa* on *C*. *albicans* WT compared to on *C*. *albicans adh1*Δ/Δ. These data were supported by the observation that *P*. *aeruginosa* produces higher levels of fluorescent siderophores on *C*. *albicans adh1*Δ/Δ compared to the WT (Fig 1E, inset). The over-representation of low iron responsive genes in both *P*. *aeruginosa* and *C*. *albicans* demonstrated that the organisms were experiencing simultaneous iron limitation but not in 5-MPCA-permissive conditions. Taken together these data are consistent with *C*. *albicans* ethanol production stimulating *P*. *aeruginosa* antagonistic 5-MPCA phenazines which affect *C*. *albicans* metabolism and ROS stress pathways and the deletion of Adh1 in *C*. *albicans* leading to increased competition for iron but not 5-MPCA production.

The lack of compensatory production of other *C*. *albicans* fermentation products such as acetate and glycerol, in the *adh1*Δ/Δ cultures (S1 Fig) led us to wonder if the loss of Adh1 may increase competition for oxygen due to an increased reliance on oxidative phosphorylation for energy generation and redox balancing. However, we did not observe an over-representation of *P*. *aeruginosa* genes regulated by Anr in response to low oxygen [79] in DEGs between *P*. *aeruginosa* grown on *C*. *albicans* WT versus *adh1*Δ/Δ (p-value = 0.25, hypergeometric test). Further, deletion of Anr did not inhibit red pigment formation in *P*. *aeruginosa* grown on *C*. *albicans* WT or promote red pigment formation in *P*. *aeruginosa* grown on *C*. *albicans adh1*Δ/Δ (Table 1).

**Table 1:** *P*. *aeruginosa* strain phenotypes for 5-MPCA-accumulation in co-culture with *C*. *albicans* WT and ethanol-induced alkaline phosphatase (AP) activity in mono-culture. AP activity was visualized in colonies on agar containing BCIP. *P*. *aeruginosa* mutants were defective in Anr, ppGpp-dependent AlgU and DksA signaling, ethanol catabolism, the kinase KinB and sigma factor VreI, which are known to influence PhoB activity, acetyl-phosphate metabolism and ethanol catabolism.

Genotype	*P*.*a*. 5MPCA on *C*. *albicans* WT	*P*.*a*. AP induction by ethanol
WT	Yes	Yes
Δ*phoB*	No	No
Δ*anr*	Yes	Yes
Δ*algU*	Yes	Yes
Δ*dksA*	Yes	Yes
Δ*relA*	Yes	Yes
Δ*relA*Δ*spoT*	Yes	Yes
Δ*kinB*	No	Yes
Δ*kinB*+*kinB*	Yes	Yes
Δ*mucB*	Yes	Yes
Δ*vreI*	n/a*	Yes
Δ*vreR*	n/a*	Yes
Δ*vreA*	n/a*	Yes
Δ*vreI*Δ*phoB*	n/a*	No
Δ*exaA*	No	No
Δ*exaA*+*exaA*	Yes	Yes
*acsA*::Tn*M*	Yes	No
Δ*ackA*	Yes	Yes
Δ*ackA*Δ*pta*	Yes	Yes
Δ*ackA*Δ*pta*Δ*phz*	No	Yes

* not available due to background strain differences

### eADAGE analysis of *P*. *aeruginosa* transcriptomes revealed additional pathways differentially active in response to ethanol in co-culture with *C*. *albicans*

**Fig 2:**
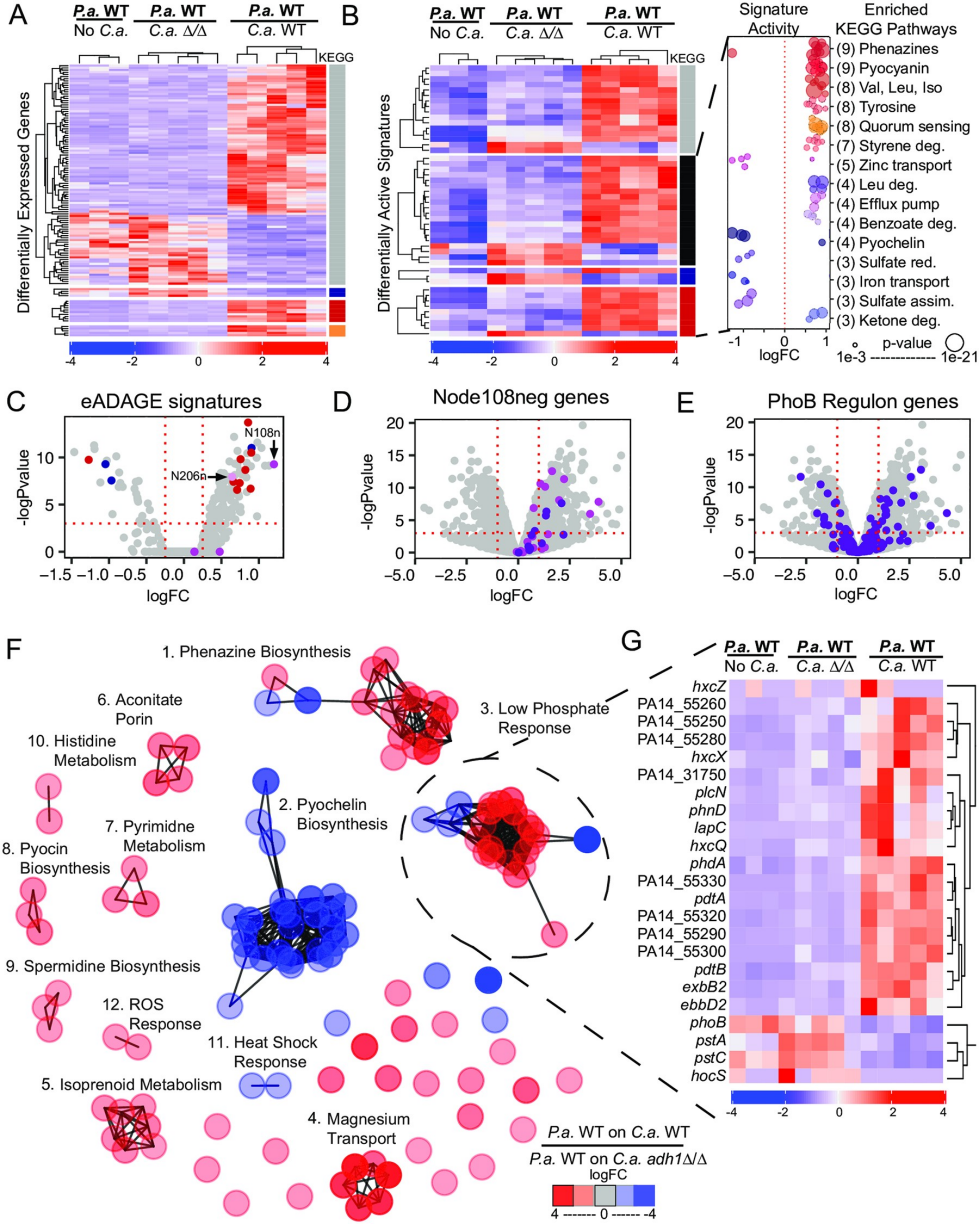
eADAGE analysis reveals a subset of the Pho regulon upregulated in *P*. *aeruginosa* (*P*.*a*.) grown on *C*. *albicans* (*C*.*a*.) WT compared to that on *C*.*a*. *adh1*Δ/Δ. A) Differentially expressed genes (DEGs) between *P*.*a*. grown alone or on *C*.*a*. WT and *C*.*a*. *adh1*Δ/Δ for 24 h. annotated with KEGG pathways (quorum sensing, orange bar; phenazine biosynthesis, red bar; and pyochelin biosynthesis, blue bar; other or none, grey). B) Differentially active eADAGE signatures (DASs) for the same samples shown in A. Signatures with over-represented KEGG pathways annotated (phenazine biosynthesis, red bar; and pyochelin biosynthesis, blue bar; other, black; or none, grey). Inset shows the fold-change for the expression all of the KEGG pathways that are over-represented among the DASs (# of DASs per KEGG pathway in parentheses); over-representation p-value shown as circle size (S2 Dataset). C) DASs with increased activity in transcriptome comparisons of *P*.*a*. grown on *C*.*a*. WT versus *adh1*Δ/Δ. Over-represented KEGG pathways and pathways of interest are colored (phenazine biosynthesis, red dots; pyochelin biosynthesis, blue dots; ethanol catabolism, N206n pink dot; the PhoB regulon, N108n violet dot). D) *P*.*a*. gene expression between co-culture with *C*.*a*. WT and *adh1*Δ/Δ, genes in N108n colored violet and genes also in the PhoB regulon in purple with DEGs cutoffs logFC > 1, FDR < 0.05 marked with red dotted lines. E) Same *P*.*a*. genes expression as D, genes in the PhoB regulon colored purple. F) Network analysis of DEGs suggest groups of DEGs have correlated patterns across eADAGE: phenazine biosynthesis (1) is inversely expressed with the low iron response (2) and coordinately upregulated with the low-phosphate response (3) upon exposure to ethanol in co-culture. Other cliques of DEGs participate in shared biological pathways. Descriptions of all cliques in S1 Table. G) The Pho Clique (3) contains two clades of DEGs with opposing expression patterns between *P*.*a*. grown on *C*.*a*. WT and *C*.*a*. *adh1*Δ/Δ.

While the analysis of DEGs and the KEGG pathways over-represented therein provided insight into key *P*. *aeruginosa*–*C*. *albicans* interactions, only 19 of the 120 *P*. *aeruginosa* strongly DEGs (|logFC| > 2, FDR < 0.01) fell within the three statistically over-represented KEGG pathways: QS (orange bar), phenazine biosynthesis (red bar) and pyochelin biosynthesis (blue bar) (Fig 2A). To look for additional processes that were affected by *C*. *albicans* ethanol production, we further identified patterns in the RNA-Seq data using eADAGE, a denoising autoencoder-based tool [53, 63, 64]. In eADAGE analysis, the activities of previously-defined gene expression signatures are calculated as weighted sums of normalized gene expression values where gene weights are unique to each signature [64]. Using eADAGE, we found 48 differentially active signatures (DASs) in *P*. *aeruginosa* grown on *C*. *albicans* WT versus *P*. *aeruginosa* grown on *C*. *albicans adh1*Δ/Δ (S2 Dataset).

As predicted by the DEGs (Fig 2A), there were multiple DASs in which phenazine biosynthesis and pyochelin biosynthesis KEGG pathways were over-represented (Fig 2B, red and blue bars respectively). The detection of multiple signatures enriched in these pathways was expected based on the presence of similar signatures that are redundant in some contexts but discriminating in others [53]. Thirty-two of the DASs (Fig 2B, black bar) had over-representations of several other KEGG pathways, and 16 DASs showed no over-representation of any KEGG pathway (Fig 2B, grey bar). Pathways over-represented in DASs included amino acid metabolism, styrene metabolism and zinc uptake (Fig 2B inset, S2 Dataset). Notably, one differentially active signature, Node206neg (N206n), contained ethanol catabolism genes (Fig 2C, pink dot). The signature with the largest increase in activity, Node108neg (N108n) (Fig 2C, violet dot) was not enriched in any KEGG pathway. However, we had previously identified Node108neg (N108n) as significantly more active in low-phosphate media than phosphate replete media across the compendium of gene expression data on which eADAGE was trained [53]. Therefore, upon identifying Node108neg as the most activated eADAGE signature in response to *C*. *albicans* ethanol production in co-culture, we further investigated the genes within Node108neg and their connection to the *P*. *aeruginosa* low-phosphate response.

### eADAGE analysis suggested *P*. *aeruginosa* PhoB up-regulated genes in response to *C*. *albicans* ethanol production

In the most upregulated eADAGE signature, Node108neg, the set of PhoB-regulated genes (i.e. the PhoB regulon) was significantly over-represented (hypergeometric test: p = 5.9x10^-9^). The PhoB regulon has been extensively defined through rigorous experimental methods including mutant transcriptomics, motif analysis and chromatin immunoprecipitation assays [55]. Of the 32 genes in Node108neg, 11 were also in the PhoB regulon (Fig 2D) and they all increased in expression in *P*. *aeruginosa* grown on *C*. *albicans* WT compared to on *adh1*Δ/Δ, but the Pho regulon defined in Bielecki *et al*. [55] was heterogeneously expressed overall (Fig 2E). We more closely examined DEGs in the context of eADAGE signatures in order understand how changes in signature activities embodied the *P*. *aeruginosa* response to *C*. *albicans* ethanol production and whether *P*. *aeruginosa* gene expression changes between growth on *C*. *albicans* WT and on *adh1*Δ/Δ signaled a low-phosphate response.

We visualized relationships among DEGs in the eADAGE gene-gene network. The full gene-gene network consists of the 5,549 *P*. *aeruginosa* genes used to create the eADAGE model as vertices with similarities in transcriptional patterns as weighted edges (shorter edges represent higher Pearson correlations between gene weights across all signatures in the eADAGE model) [53, 63, 64]. Here we show strongly DEGs (logFC > 2, FDR < 0.01) between *P*. *aeruginosa* grown on *C*. *albicans* WT and on *adh1*Δ/Δ connected by edges whose weights are drawn from the full gene-gene network (edge cutoff ± 0.5) (Fig 2F). DEGs fell into cliques (S1 Table) when visualized as a sub-network within the eADAGE gene-gene network (Fig 2F). The three largest cliques contained genes relevant to the biological processes of phenazine biosynthesis (clique 1, 17 genes), pyochelin biosynthesis (clique 2, 29 genes), and the low-phosphate response (clique 3, 23 genes) (Fig 2F). Other cliques contained genes involved in magnesium flux across the membrane (clique 4), isoprenoid catabolism (clique 5), aconitate porins (clique 6), pyrimidine metabolism (clique 7), pyocin biosynthesis (clique 8), spermidine biosynthesis (clique 9), histidine metabolism (clique 10), the heat shock response (clique 11), and the ROS stress response (clique 12). Most notably, many genes within clique 3, which were related to the low-phosphate response, were also in Node108neg.

Genes within clique 3, which were enriched in the DAS Node108neg, clustered into two groups: four genes were more highly expressed in *P*. *aeruginosa* grown on *C*. *albicans adh1*Δ/Δ (*phoBR* and *pstABC*) and nineteen were more highly expressed in *P*. *aeruginosa* grown on *C*. *albicans* WT (Fig 2G). The group of *P*. *aeruginosa* genes that were more highly expressed on *C*. *albicans* WT included those that encode the Hxz type II secretion system (PA14_55450, PA14_55460) and its substrate enzyme the phosphatase *lapC* [80], the TonB-dependent transporter ExbB2/D2, the phosphatase extracellular alkaline phosphatase PhoA, and the secreted phospholipase PlcN. The transcriptomes of *P*. *aeruginosa* grown alone were also analyzed: *P*. *aeruginosa* did not have an activated low-phosphate response in mono-culture as transcripts associated with the PhoB regulon were very low and not different from levels in *P*. *aeruginosa* grown with *C*. *albicans adh1*Δ/Δ (S3 Dataset).

### The *P*. *aeruginosa* low-phosphate response was activated in co-culture with *C*. *albicans* WT but not *adh1*Δ/Δ

**Fig 3:**
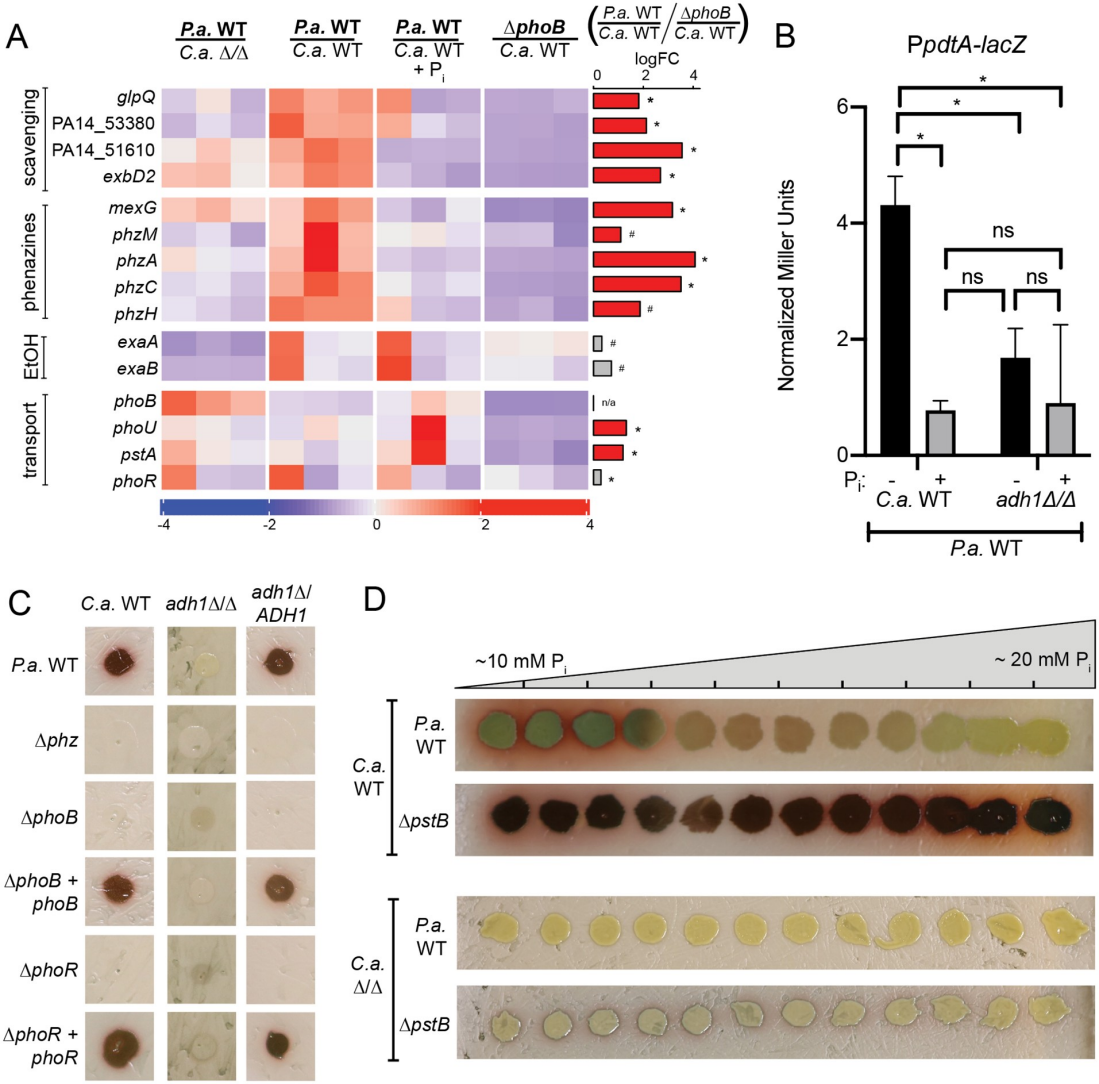
*C*. *albicans* (*C*.*a*.) WT induced PhoB-regulated genes in *P*. *aeruginosa* (*P*.*a*.) compared to *C*.*a*. *adh1*Δ/Δ leading to 5-MPCA production as indicated by red pigment formation. A) Expression of *P*.*a*. genes involved in phosphate scavenging, phenazines, ethanol catabolism (EtOH) and inorganic phosphate transport were measured by NanoString (codesetPAV5) from cells grown with *C*.*a*. *adh1*Δ/Δ, *C*.*a*. WT or *C*.*a*. WT grown on YPD+10 mM phosphate (P_i_). Expression values are normalized to loading controls and housekeeping genes, z-score scaled by gene. Right-hand bar plot shows logFC between *P*.*a*. WT and *P*.*a*. Δ*phoB* on *C*.*a*. WT. The bar is colored red if expression is PhoB-dependent (logFC *P*.*a*. WT / *P*.*a*. Δ*phoB* > 1, FDR < 0.05), else grey. *, FDR < 0.05, #, FDR > 0.05. B) Beta-galactosidase activity indicative of expression of a *pdtA*-*lacZ* promoter fusion in *P*.*a*. WT in *P*.*a*. grown with *C*.*a*. WT or *C*.*a*. *adh1*Δ/Δ in the absence or presence of P_i_ supplementation. Values are normalized to paired Δ*phoB* baselines. *,p<0.05 by ANOVA with Tukey’s multiple comparison test (n = 3). C) Red 5-MPCA derivatives produced in co-culture by *P*.*a*. WT, Δ*phoB*, Δ*phoR*, and their complemented derivatives on *C*.*a*. WT, *adh1*Δ/Δ, and *adh1*Δ/*ADH1*. D) Red 5-MPCA-derivatives produced by *P*.*a*. WT and *P*.*a*. Δ*pstB* in co-culture with *C*.*a*. WT (top) or *C*.*a*. *adh1*Δ/Δ (bottom) over a gradient of phosphate concentrations.

We confirmed that some PhoB-regulated transcripts were higher in *P*. *aeruginosa* co-cultured with *C*. *albicans* WT versus *adh1*Δ/Δ using the NanoString multiplex RNA analysis method. Seventy-five transcripts were monitored, and data were normalized to six housekeeping genes as described in the Methods section. Again, we found that genes encoding phosphate scavenging enzymes were more highly expressed in *P*. *aeruginosa* on *C*. *albicans* WT than on *adh1*Δ/Δ (Fig 3A, top section), while phosphate transport-associated genes (*phoR*, *phoB*, *pstA* and *phoU*) were inversely regulated (Fig 3A, bottom section). Consistent with the phenotypic data, genes involved in phenazine biosynthesis (*phzM*, *phzA*, *phzC*, and *phzH*) and efflux (*mexG*) were higher on *C*. *albicans* WT than on *C*. *albicans adh1*Δ/Δ as well (Fig 3A, second section).

As PhoB activity is controlled by phosphate levels [81], we assessed the effects of phosphate supplementation in co-culture. The addition of 10 mM potassium phosphate to the medium underlying the co-cultures resulted in a decrease in the expression levels of PhoB regulated genes similar to the deletion of *P*. *aeruginosa phoB* (Fig 3A top section). The histogram to the right of Fig 3A shows the mean signal of PhoB-dependence (*P*. *aeruginosa* WT on *C*. *albicans* WT / *P*. *aeruginosa* Δ*phoB* on *C*. *albicans* WT). *P*. *aeruginosa* ethanol catabolism genes *exaA* and *exaB* were also upregulated in *P*. *aeruginosa* on *C*. *albicans* WT than on *adh1*Δ/Δ. The expression of these genes was unaffected by phosphate supplementation or deletion of *phoB*.

PhoB activity has been shown to affect phenazine production previously [39]. We found that the increased expression of *P*. *aeruginosa* genes involved in phenazine biosynthesis and efflux (*phzM*, *phzA*, *phzC*, *phzH*, and *mexG*) on *C*. *albicans* WT versus on *adh1*Δ/Δ was suppressed by supplementation with 10 mM phosphate and was not observed in *P*. *aeruginosa* Δ*phoB* mutant. As we and others have shown the antifungal effects of phenazines [25, 27, 30], these data provide a strong link between phosphate availability and antagonism in these co-cultures.

To accompany the transcript abundance data, we assessed PhoB-dependent promoter activity using a *P*. *aeruginosa pdtA* promoter fusion strain [41]. We found that PhoB-dependent *pdtA* promoter activity decreased in response to 10 mM phosphate in co-culture with *C*. *albicans* WT (Fig 3B). In concert, transcript abundance and promoter activity data suggested that in co-culture, the bioavailability of phosphate modulated PhoB activity.

### PhoB was necessary for 5-MPCA-derived red pigment accumulation in *P*. *aeruginosa*—*C*. *albicans* co-culture

Given the dependence on PhoB for the expression of phenazine biosynthesis genes, we determined if PhoB was necessary for the accumulation of 5-MPCA-derived red pigment in co-culture with *C*. *albicans* WT. *P*. *aeruginosa* Δ*phoB* did not support the accumulation of the 5-MPCA-derivative as indicated by the lack of red pigment in co-culture with ethanol-producing *C*. *albicans* WT, and this was restored by chromosomal complementation with a wild-type copy of *phoB* in *P*. *aeruginosa*, provided that *C*. *albicans* had a functional *ADH1* gene (Fig 3C). Phenazine production was also dependent on PhoR, the known regulatory kinase of PhoB, and the Δ*phoR* phenotype was complemented by a wild-type copy of *phoR* expressed on an extrachromosomal plasmid.

We further demonstrated that PhoB activity was required for 5-MPCA production by phosphate supplementation to the medium prior to co-culture inoculation. Decreased 5-MPCA-derived red pigment accumulation in *P*. *aeruginosa* WT co-culture with *C*. *albicans* WT was seen across a phosphate gradient plate (Fig 3D, top). The ability of phosphate to quench red pigment formation suggested that co-culture biofilms may deplete the phosphate to below a threshold of activation for the *P*. *aeruginosa* PhoB response. Increased PhoB activity was sufficient to overcome suppression by phosphate supplementation as a mutant lacking the phosphate transport ATPase PstB with constitutively active PhoB, continued to produce 5-MPCA-derived red pigment even at high phosphate concentrations (Fig 3D). As expected, *P*. *aeruginosa* WT did not form any 5-MPCA-derived pigment in co-culture with *C*. *albicans adh1*Δ/Δ at any phosphate concentration tested. Surprisingly, *P*. *aeruginosa* Δ*pstB* grown on *adh1*Δ/Δ only slightly rescued 5-MPCA-derived red pigment formation (Fig 3D). Therefore, a difference in the ability of *C*. *albicans* WT and *adh1*Δ/Δ to deplete phosphate was not fully responsible for differences in *P*. *aeruginosa* PhoB-dependent red pigment formation, but rather *C*. *albicans* Adh1 activity provided an additional stimulus, such as ethanol, that created conditions conducive to PhoB-regulated *P*. *aeruginosa* antifungal phenazine production.

### Ethanol was sufficient to activate PhoB at permissive phosphate concentrations

**Fig 4:**
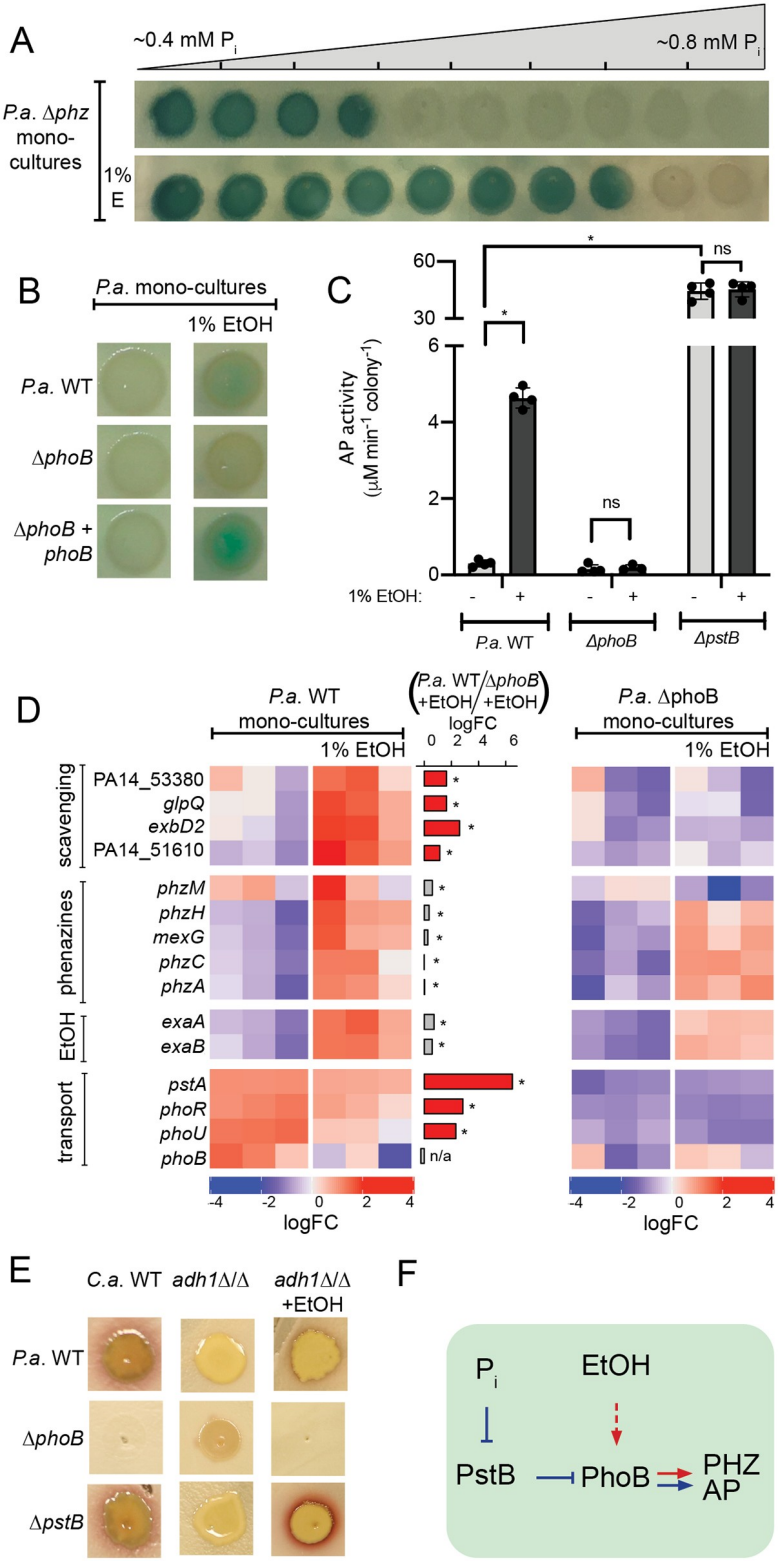
Ethanol (EtOH) induced PhoB activity in *P*. *aeruginosa* (*P*.*a*.) mono-culture. A) Alkaline phosphatase (AP) activity visualized by blue color derived from cleavage of BCIP in MOPS medium,0.2% glucose, with a gradient of phosphate (P_i_) in the absence and presence of ethanol. The *P*.*a*. Δ*phzA1-G1/A2-G2* strain was used to eliminate color differences due to phenazine production. B) AP activity in the absence and presence of 1% ethanol (E or EtOH) at 0.7 mM P_i_ for *P*.*a*. wild type, Δ*phoB* and the Δ*phoB* mutant complemented with a wild-type copy of *phoB* integrated at the native locus. C) AP activity in cells from colony biofilms grown as in B was measured using the colorimetric substrate pNPP for *P*.*a*. WT, Δ*phoB*, and Δ*pstB*. *,p<0.01 by ANOVA with Tukey’s multiple comparison test (n ≥ 3). D) Transcripts within the PhoB regulon involved in P_i_ scavenging and transport and genes involved in phenazine production and EtOH catabolism in *P*.*a*. WT and Δ*phoB* cells grown in the absence and presence of EtOH by Nanostring (codeset PAV5). Expression values normalized to loading controls and housekeeping genes, z-score scaled by gene. Middle bar plot shows log_2_ fold-change (logFC) between *P*.*a*. WT and *P*.*a*. Δ*phoB* on MOPS+1%EtOH. The bar is colored red if expression is PhoB-dependent (logFC *P*.*a*. WT / *P*.*a*. Δ*phoB* > 1, FDR < 0.05) else grey. *,FDR < 0.05, #,FDR > 0.05. E) *P*.*a*. WT, Δ*phoB* and Δ*pstB* grown on *C*.*a*. WT, or *C*.*a*. *adh1*Δ/Δ in the absence or presence of 1% exogenous EtOH. F) P_i_ and EtOH are additive stimuli that promote PhoB-dependent expression of AP and phenazine biosynthesis (PHZ).

To determine if ethanol could contribute to the stimulation of PhoB in *P*. *aeruginosa*–*C*. *albicans* co-culture, we tested whether it was sufficient to stimulate PhoB in *P*. *aeruginosa* mono-culture. To do so, we monitored PhoB-regulated alkaline phosphatase (AP) activity by the conversion of 5-bromo-4-chloro-3-indolylphosphate (BCIP) into a colorimetric substrate according to published methods [82–84]. On MOPS (3-morpholinopropane-1-sulfonic acid) buffered minimal medium after growth for 16 h at 37°C, *P*. *aeruginosa* Δ*phz*, wherein all blue coloration can be attributable to BCIP conversion and not phenazines, AP was detected up to approximately 0.55 mM phosphate in the absence of ethanol (Fig 4A). With the addition of 1% ethanol to the medium, *P*. *aeruginosa* showed AP activity at higher phosphate concentrations, up to approximately 0.73 mM phosphate (Fig 4A). This induction was also seen in *P*. *aeruginosa* WT, but not Δ*phoB*, at 0.7 mM phosphate on single concentration plates, and was restored upon complementation with a WT copy of *phoB* (Fig 4B). Quantification of AP activity under these conditions showed a 4-fold increase in the presence of 1% ethanol for *P*. *aeruginosa* WT, only trace AP activity for *P*. *aeruginosa* Δ*phoB* and hyperactivity in Δ*pstB* (Fig 4C).

To determine if PhoB was acting upon the same targets upon ethanol supplementation in mono-culture as shown for co-culture, we monitored the expression of PhoB targets using the same Nanostring codeset described earlier. *P*. *aeruginosa* WT and Δ*phoB* were grown in mono-culture on MOPS minimal medium at 0.7 mM phosphate with and without 1% ethanol. The same subset of PhoB-regulated genes indicated in co-culture expression analysis increased in *P*. *aeruginosa* WT upon ethanol supplementation, including: *phoA*, an alkaline phosphatase; *phnD*, a phosphonate transporter; *glpQ*, a glycerophosphoryl diester phosphodiesterase (Fig 4D, top section). In mono-culture, when ethanol was supplemented into the medium, we saw an increase in phenazine biosynthesis genes however, unlike in co-culture, the magnitudes of their fold-changes between *P*. *aeruginosa* WT and Δ*phoB* was not as high as those of other PhoB-regulated genes suggesting their expression was stimulated by PhoB-independent factors (Fig 4D, second panel). The set of PhoB-regulated genes whose expression was heterogenous and did not trend upward in co-culture were also not activated in mono-culture upon ethanol supplementation (Fig 4D, bottom panel). Stimulation of PhoB by ethanol in mono-culture suggests that ethanol and low phosphate were stimuli that could have both activated PhoB in co-culture. Activation of PhoB in the presence of ethanol may be indicative of physiological changes that increased phosphate utilization by *P*. *aeruginosa*. We noted that mono-culture colony biofilms of *P*. *aeruginosa* grown on medium with 1% ethanol had no difference in CFUs or cell culture density upon comparison to the ethanol-free condition (S3 Fig). This suggests that ethanol may increase phosphate utilization or decrease phosphate availability without affecting culture growth.

In light of the recent characterization of the *P*. *aeruginosa* response to exogenous ethanol [31] that showed ppGpp, synthesized by RelA and SpoT, DksA, and AlgU mounted a transcriptional response to ethanol, we assessed mutants for ethanol-induced PhoB activation in co-culture by monitoring red pigment accumulation and in mono-culture via BCIP assay. We determined that these genes were not necessary to induce PhoB activity in response to ethanol (Table 1). We also ruled out roles for known mechanisms of alternative PhoB activation including contributions of the non-canonical histidine kinase KinB [53] in both co- and mono- culture and the extra-cytoplasmic function (ECF) sigma factor VreI [37, 41] in monoculture, but further investigation is required to determine if VreI played a role in regulating 5-MPCA production in co-culture (Table 1).

We also tested the role of ethanol catabolism in PhoB activation in both mono- and co-culture. Mutants in the ExaA-dependent pathway for ethanol catabolism through acetate including *exaA* and *acsA* did not show increases in AP activity in response to 1% ethanol (Table 1) indicating that ethanol catabolism was essential for PhoB activation in mono-culture. We hypothesized that ethanol catabolism led to increased levels of acetyl phosphate, a non-canonical phosphate donor for transcription factors including PhoB, [85–88] but neither acetyl phosphate biosynthesis mediated by AckA nor catabolism by Pta was necessary for ethanol-induced PhoB activity (Table 1). Mutants defective in the ExaA-dependent ethanol catabolic pathway showed only weak 5-MPCA production on *C*. *albicans* lawns and the phenotype could be complemented (Table 1). Together these data suggest that ethanol catabolism plays a role in PhoB activation, perhaps by increasing phosphate utilization, and that additional pathways for ethanol catabolism may be present in co-culture conditions. Further investigation is required to determine the mechanism for ethanol induction of PhoB activity, but these results demonstrate ethanol stimulation and PhoB activation participated in non-linear but intersecting pathways.

### Low phosphate and ethanol were additive stimulants for PhoB-mediated red pigment formation

We tested whether ethanol stimulation and canonical PhoB de-repression had additive effects on 5-MPCA-derived red pigment formation in *P*. *aeruginosa–C*. *albicans* co-culture. On non-ethanol producing *C*. *albicans adh1*Δ/Δ, the constitutive PhoB activity in *P*. *aeruginosa* Δ*pstB* was not sufficient to stimulate red pigment to the extent of that on ethanol-producing *C*. *albicans* WT (Fig 4E). To determine if repressive stimuli that were present in *C*. *albicans adh1*Δ/Δ, but not WT, co-cultures had a negative effect on *P*. *aeruginosa* red pigment formation or if the absence of ethanol was the reason for decreased 5MPCA production, we performed an ethanol supplementation experiment. Strikingly, we found that the addition of 1% ethanol to *C*. *albicans* a*dh1*Δ/Δ lawns increased red pigment formation in *P*. *aeruginosa* Δ*pstB* but not in *P*. *aeruginosa* WT or Δ*phoB* (Fig 4E). This revealed that in addition to PhoB-independent effects of ethanol on phenazines production, PhoB activation both through the canonical signaling pathway and by means of ethanol stimulation were necessary for 5-MPCA-derived red pigment formation in co-culture (proposed model in Fig 4F).

### The *C*. *albicans* low-phosphate response was more active in *adh1*Δ/Δ than in WT

**Fig 5:**
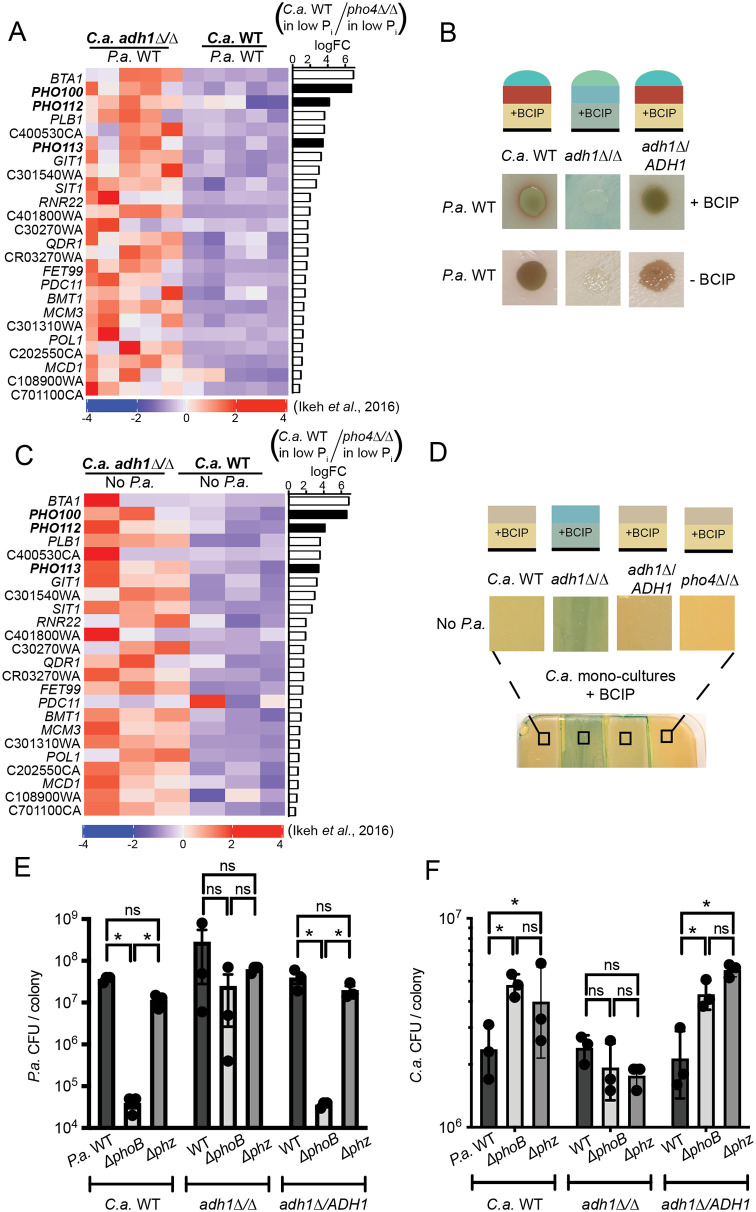
The *C*. *albicans* (*C*.*a*.) *adh1*Δ/Δ has increased expression of the Pho4-mediated low-phosphate response. A) Previously characterized Pho4-regulated genes [89] including a phospholipase and two phosphatases (black bars) were more highly expressed in *C*.*a*. *adh1*Δ/Δ than *C*.*a*. WT in *P*.*a*. co-cultures (data shown as z-scores of TPM). Pho4-dependence is shown in the right-hand bar plot as log_2_FC *C*.*a*. WT/*C*.*a*. *pho4*Δ/Δ using data from [89]. B) Analysis of phosphatase activity in *C*.*a*. WT, *C*.*a*. *adh1*Δ/Δ or the complemented strain *adh1*Δ/*ADH1* using the colorimetric phosphatase substrate BCIP. C) The same Pho4-regulated genes as shown in A in mono-cultures. Right-hand bar plot shows Pho4-dependence as in A. D) More phosphatase activity was observed in *C*.*a*. *adh1*Δ/Δ than in strains with *ADH1* in in mono-culture. As predicted, phosphatase activity is not evident in the *C*.*a*. *pho4*Δ/Δ strain. Phosphatase activity visualized via BCIP as in B. E) CFUs of *P*.*a*. WT, Δ*phoB* or Δ*phz* after co-culture for 72 h with *C*.*a*. WT, *C*.*a*. *adh1*Δ/Δ or *adh1*Δ/*ADH1*. *,p<0.01 by ANOVA with Dunnett’s multiple comparisons test (n ≥ 3). F) CFUs of *C*.*a*. from the same samples analyzed in E. *,p<0.01 Student’s t-test adjusted for multiple comparisons by Holm-Sidak method (n ≥ 3).

Given the differences between *P*. *aeruginosa* PhoB activity in co-culture with *C*. *albicans* WT and *adh1*Δ/Δ, we sought to understand if *C*. *albicans* also experienced differences in phosphate availability by examining its low-phosphate response. In *C*. *albicans*, low phosphate induces activity of the transcription factor Pho4 which regulates genes involved in phosphate acquisition as well as the homeostasis of cations (e.g. iron), tolerance of stresses including ROS and arsenic, and fitness in murine models [89–93]. Pho4 regulates 133 genes that were previously identified by Ikeh *et al*. using transcriptomics as differentially expressed both between *C*. *albicans* WT and *pho4*Δ/Δ in low phosphate and between *C*. *albicans* WT in high and low phosphate [89] (see S4 Dataset for gene list). We found that Pho4-regulated genes were over-represented in DEGs between co-cultures of *C*. *albicans* WT with *P*. *aeruginosa* and *C*. *albicans adh1*Δ/Δ with *P*. *aeruginosa* (|logFC| > 1, p < 0.05) (hypergeometric test p = 1.9x10^-3^). Of the top ten most differentially expressed genes between *C*. *albicans* WT and *pho4*Δ/Δ in low phosphate as determined by Ikeh *et al*. [89], seven were also strongly differentially expressed (logFC > 2) in co-cultures of *C*. *albicans* WT with *P*. *aeruginosa* compared to *C*. *albicans adh1*Δ/Δ with *P*. *aeruginosa* including a secreted phospholipase (*PHO100*) and two secreted phosphatases (*PHO112* and *PHO113*) (Fig 5A, black bars). Given the strong differential expression of these phosphatases, we assessed phosphatase activity via BCIP assay as done for *P*. *aeruginosa*. Consistent with the transcriptional data, higher phosphatase activity was observed in *C*. *albicans adh1*Δ/Δ compared to *C*. *albicans* WT or the complemented strain *adh1*Δ/*ADH1* in the presence of *P*. *aeruginosa* (Fig 5B).

To determine if the activation of the *C*. *albicans* low-phosphate response in *adh1*Δ/Δ in co-culture was a consequence of competition for phosphate with *P*. *aeruginosa* we examined *C*. *albicans* gene expression and phosphatase activity in mono-culture. We found that the same Pho4-regulated *C*. *albicans* genes that were more highly expressed in *C*. *albicans adh1*Δ/Δ than in *C*. *albicans* WT in co-culture with *P*. *aeruginosa* were also more highly expressed in *C*. *albicans adh1*Δ/Δ when each strain was grown alone (Fig 5C). Consistent with the increased expression of phosphatase genes, we also observed higher phosphatase activity in *C*. *albicans adh1*Δ/Δ cultures when compared to *C*. *albicans* WT or the complemented strain *adh1*Δ/*ADH1* in mono-culture using the BCIP assay (Fig 5D). For reference, we assayed *C*. *albicans pho4*Δ/Δ and found that the low levels of phosphatase activity seen for *C*. *albicans* WT and *adh1*Δ/*ADH1* were not evident in *pho4*Δ/Δ indicating that that phosphatase activity was Pho4-dependent (Fig 5D). The high Pho4 response in *C*. *albicans adh1*Δ/Δ, evident even in mono-culture, suggested that Adh1 activity impacts *C*. *albicans* phosphate access, phosphate requirements or Pho4 regulation. By analyzing changes in gene expression between *C*. *albicans adh1*Δ/Δ and WT compared to their respective mono-culture conditions, we did not find over-representation of the Pho4 regulon in co-culture conditions compared to *C*. *albicans* only cultures (hypergeometric test: p > 0.05) (S1 Dataset). Since phosphatases produced by *C*. *albicans* as part of its low-phosphate response are secreted, we hypothesized that their production could affect *P*. *aeruginosa* in co-culture, perhaps by providing access to phosphate liberated from macromolecules and this model is discussed further below.

### In co-culture with ethanol-producing *C*. *albicans*, *P*. *aeruginosa* PhoB plays independent roles in phosphate scavenging and phenazine-mediated antagonism

PhoB was important for *P*. *aeruginosa* growth in co-culture as Δ*phoB* formed fewer CFUs than did *P*. *aeruginosa* WT on *C*. *albicans* WT (Fig 5E). Notably, a comparable number of *P*. *aeruginosa* CFUs were recovered from *P*. *aeruginosa* WT and Δ*phz* in co-culture suggesting that the lack of phenazines was not a major reason for decreased CFU formation in Δ*phoB* (Fig 5E). The defect in CFU formation by Δ*phoB* when compared to WT or Δ*phz* held true on *C*. *albicans* with a complemented copy of *ADH1*. On the *C*. *albicans adh1*Δ/Δ mutant, however, *P*. *aeruginosa* CFUs were similar for *P*. *aeruginosa* WT, Δ*phoB*, and Δ*phz* suggesting that in the absence of *C*. *albicans* Adh1 activity, *P*. *aeruginosa* PhoB, and its roles in phosphate acquisition or phenazine production, were no longer necessary for fitness. This finding is consistent with the model that elevated phosphatase production by the *C*. *albicans adh1*Δ/Δ may eliminate the need for *P*. *aeruginosa* to produce phosphatases (Fig 5B and 5D).

*C*. *albicans* CFUs were enumerated from the same co-cultures. Consistent with previous reports on the antifungal properties of the phenazine 5-MPCA [25, 27], *C*. *albicans* had lower CFUs upon co-culture with *P*. *aeruginosa* WT compared to when co-cultured with Δ*phoB* or Δ*phz* (Fig 5F). In *C*. *albicans adh1*Δ/Δ co-cultures which did not support *P*. *aeruginosa* PhoB-dependent phenazine production (Fig 3), *C*. *albicans* CFUs were not different between co-cultures with *P*. *aeruginosa* WT, Δ*phoB* or Δ*phz*.

### *P*. *aeruginosa* and *C*. *albicans* asynchronously activated their low-phosphate responses dependent on *C*. *albicans* Adh1

**Fig 6:**
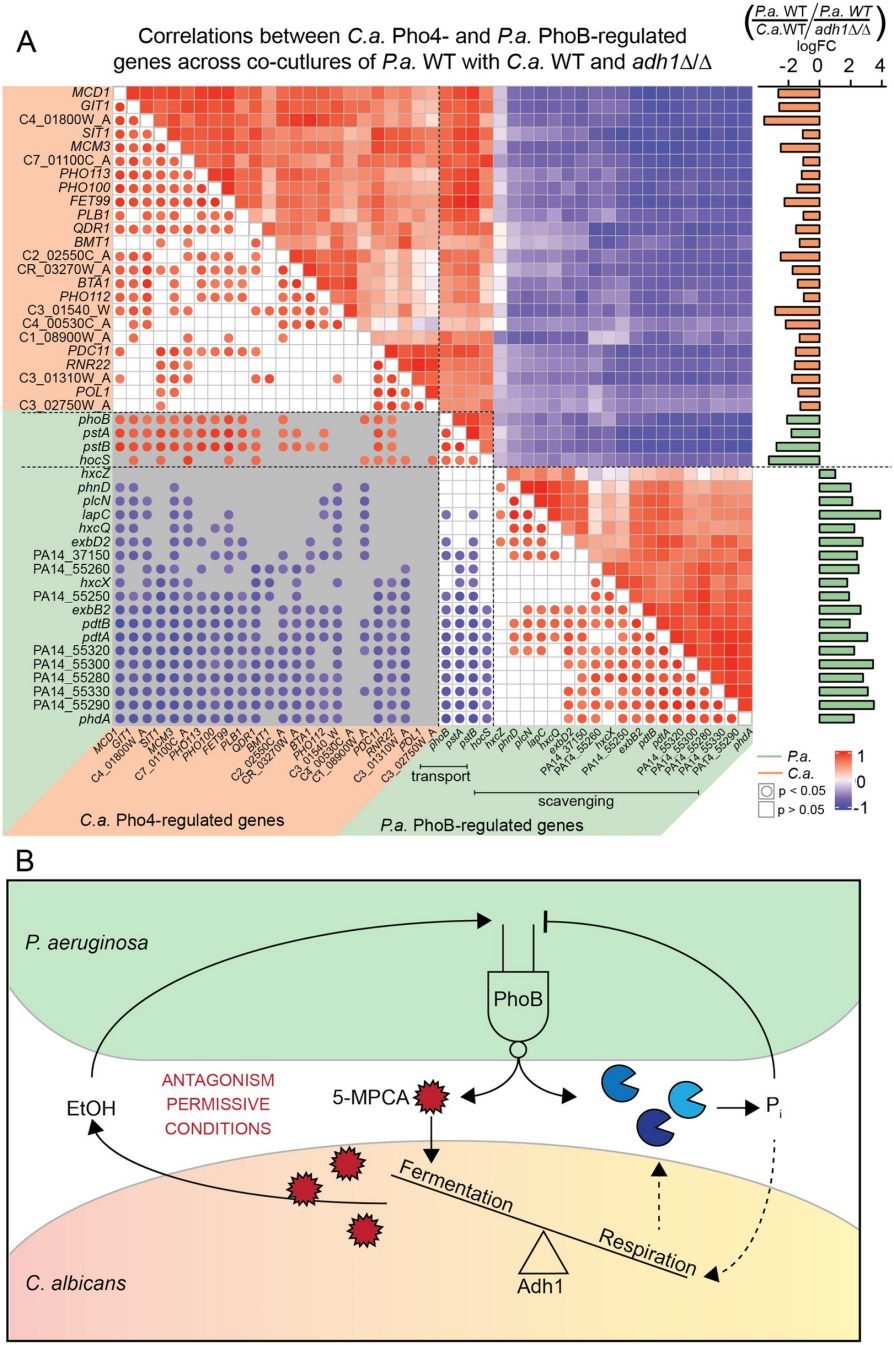
*P*. *aeruginosa* (*P*.*a*.) and *C*. *albicans* (*C*.*a*.) asynchronously active low-phosphate responses. A) Pearson correlation analysis between *P*.*a*. (green annotations) and *C*.*a*. (orange annotations) low-phosphate responsive genes from co-cultures of *P*.*a*. WT with either *C*.*a*. WT or *adh1*Δ/Δ. Inverse cross-species correlations between *P*.*a*. PhoB- and *C*.*a*. Pho4-regulated genes were apparent. Log_2_FC (p<0.05) between *P*.*a*. with either *C*.*a*. WT or *C*.*a*. *adh1*Δ/Δ is shown in the right-hand bar plot. Lower half of correlogram shows which correlations are significant (filled circles) and indicates correlation values by color intensity relative to scale. Same species comparisons have white backgrounds and cross-species correlations have grey backgrounds. B) Model of PhoB activity in *P*.*a*.*-C*.*a*. co-cultures. PhoB mediates the conditional production of the antagonistic, antifungal phenazine 5-MPCA in response to low phosphate and fungal ethanol production.

While *P*. *aeruginosa* had higher activity of its low-phosphate responsive regulator PhoB and phosphatase production in the presence of ethanol-producing *C*. *albicans* WT (Figs 2G, 3A and 3B), *C*. *albicans* had higher activity of its low-phosphate responsive transcription factor Pho4 and higher phosphatase levels in *C*. *albicans adh1*Δ/Δ (Fig 5). To more completely examine the relationship between the *P*. *aeruginosa* and *C*. *albicans* low-phosphate responses in co-culture, we performed a cross-species correlation analysis on the dual-seq co-culture gene expression data for a subset of *P*. *aeruginosa* PhoB-regulated genes (clique 3) and a subset of *C*. *albicans* Pho4-regulated genes (those also DEGs between *C*. *albicans* WT and *adh1*Δ/Δ shown in Fig 5A). There was a striking pattern of inverse correlations between *P*. *aeruginosa* PhoB-regulated genes and *C*. *albicans* Pho4-regulated genes (Fig 6A, upper triangle). In addition to being of high magnitudes, many same-species correlations (white background) as well as cross-species correlations (grey background) were statistically significant as indicated by circles (Fig 6A, lower triangle).

The asynchronous and apparently inverse activations of the *P*. *aeruginosa* and *C*. *albicans* low-phosphate responses in co-culture, in combination with the differences in the requirement for PhoB on *C*. *albicans* WT versus *adh1Δ/Δ* (Fig 5E and 5F), suggest that the two microbes were not responding to common environmental stimuli but rather that they influenced each other. The increased phosphatase activity from *C*. *albicans adh1*Δ/Δ even in the absence of *P*. *aeruginosa* (Fig 5D) suggested that activation of the *C*. *albicans* low-phosphate response may have prevented activation of the *P*. *aeruginosa* low-phosphate response. We speculate increased phosphate availability in concert with the lack of ethanol production led to low PhoB activity in *P*. *aeruginosa* in co-culture with *C*. *albicans adh1*Δ/Δ and a consequent lack of 5-MPCA-derived accumulation.

Taken together, our results led to the model that *C*. *albicans* fermentative metabolism promoted *P*. *aeruginosa* PhoB-dependent 5-MPCA production through positive stimulation by ethanol and a competition for phosphate that lead to permissively low phosphate concentrations (Fig 6B). PhoB and phenazine production decreased *C*. *albicans* fitness (Fig 5F). Inversely, loss of Adh1 in *C*. *albicans* removed the positive effects of ethanol on *P*. *aeruginosa* and increased *C*. *albicans* Pho4 activity and phosphatase secretion which we hypothesize liberated sufficient phosphate to suppress *P*. *aeruginosa* PhoB-dependent 5-MPCA production (Fig 6B).

## Discussion

Here, we show that *P*. *aeruginosa* production of the antifungal phenazine 5-MPCA is dependent on PhoB and *C*. *albicans* ethanol production. As ethanol is a common metabolite secreted by many fermentative organisms and phosphate is a universally essential nutrient, the processes cross-regulated by ethanol and phosphate signaling in the model organisms *P*. *aeruginosa* and *C*. *albicans* may be generalizable and aid in understanding the dynamics of complex, diverse microbial communities. These results also highlight the need to consider environmental factors when characterizing microbial interactions.

While we did not elucidate the mechanism by which ethanol influences PhoB activity, we identified ExaA-based ethanol catabolism as a potential regulator of the PhoB-mediated ethanol response including 5-MPCA production [94, 95]. Ethanol induction of PhoB did not require either acetyl phosphate or ppGpp (Table 1), two pathways previously linked to ethanol catabolism [96, 31], and thus we speculate that the effects could be multifactorial and may involve conditions such as changes in NAD(P)H/NAD(P)+ ratios, the generation of acetaldehyde or acetate as metabolic intermediates, or other roles played by enzymes involved in ethanol oxidation [97]. The influence of ethanol on PhoB and phenazine production may also involve other regulators as it has been linked to PQS and RhlR signaling [36, 39, 76], and may interface with systems that respond to distinct cues such as nutrient depletion, surface signals or slow-growth states. The Pho regulon has been well characterized in terms of direct PhoB targets [55], but has also been shown to include additional indirect targets across various low-phosphate media [44].

The constant utilization and solubilizing of phosphate in dynamic co-cultures creates an environment which is quite different from conditions in the low-phosphate media in which the canonical low-phosphate response was defined. While the non-canonical PhoB response reported here had an unusual divergence in the expression of the *phoBR* genes themselves and phosphate transport genes from the expression of other PhoB targets, eADAGE was able to identify this signal. The 600 gene expression patterns observed by eADAGE through its unsupervised training on over 1,000 publicly available *P*. *aeruginosa* gene expression profiles found signature Node108neg, which contained this non-canonical subset of the PhoB regulon activated in co-culture and by ethanol. Other signatures in eADAGE, such as Node164pos, contained the canonical PhoB regulon with both *phoBR* and the *pst* genes along with genes encoding phosphate scavenging enzymes. Our analysis by eADAGE also facilitated the development of other hypotheses that can be tested in the future. We observed enrichment of genes involved in leucine catabolism which may be indicative of *P*. *aeruginosa* response to *C*. *albicans*-produced farnesol [18, 28, 98, 99]. Other interesting pathways that emerged in the eADAGE analysis were those for sulfur assimilation, zinc transport, and iron uptake, and co-culture conditions may provide an opportunity to understand the connections between these pathways and their roles in stress responses and virulence factor production. This study highlights the power of using unsupervised machine learning methods, in conjunction with a compendium of versatile conditions, to identify higher order gene expression patterns that are not evident in linear correlation-based analyses and have not yet been manually annotated or systematically described.

The intersection of ethanol and phosphate signaling may serve to limit *P*. *aeruginosa* production of 5-MPCA and other antifungal products to when ethanol-producing species are nearby and *P*. *aeruginosa* is phosphate-limited. This type of two-factor regulation manages a dynamic relationship between these two opportunistically pathogenic model organisms. These findings also suggest that the dynamics of phosphate sensing, transport and enzymatic release by one organism can influence the behavior of other microbes in complex ways, and these relationships must be considered in the prediction of outcomes in relationships with conditional antagonism.

## Materials and methods

### Strains and growth conditions

Bacterial strains and plasmids used in this study are listed in S2 Table. Bacteria were maintained on LB (lysogeny broth) with 1.5% agar [100]. Yeast strains were maintained on YPD (yeast peptone dextrose) with 2% agar. Where stated, ethanol (200-proof) was added to the medium (liquid or molten agar) to a final concentration of 1% and BCIP (Sigma-Aldrich #1158002001, stock solution of 60 μg/mL dimethylformamide) to a final concentration of 6 ng/L. Planktonic cultures were grown on roller drums at 37°C for *P*. *aeruginosa* and at 30°C for *C*. *albicans*.

### Construction of in-frame deletion, complementation and expression plasmids

Construction of plasmids, including in-frame deletion and complementation constructs, was completed using yeast cloning techniques in *Saccharomyces cerevisiae* as previously described [101] or Gibson assembly [102, 103]. Primers used for plasmid construction are listed in S3 Table. In-frame deletion and single copy complementation constructs were made using the allelic replacement vector pMQ30 [101]. Promoter fusion constructs were made using a modified pMQ30 vector with *lacZ*-*GFP* fusion integrating at the neutral *att* site on the chromosome. The *pdtA* promoter region 199 bp upstream of the transcriptional start site (that included a PhoB binding site) was amplified from WT *P*. *aeruginosa* PA14 gDNA using the Phusion High-Fidelity DNA polymerase with primer tails homologous to the modified pMQ30 ATT KI vector containing tandem *lacZ-gfp* reporter genes. All plasmids were purified from yeast using Zymoprep Yeast Plasmid Miniprep II according to manufacturer's protocol and transformed into *E*. *coli* strain S17/λpir by electroporation. Plasmids were introduced into *P*. *aeruginosa* by conjugation and recombinants were obtained using sucrose counter-selection. Genotypes were screened by PCR and plasmid constructs were confirmed by sequencing.

### Co-culture and mono-culture colony biofilms

Co-cultures were inoculated first with 300 μl of a *C*. *albicans* culture in YPD grown for 16 then diluted in dH_2_O to OD_600_ = 5, which was bead spread onto YPD plates. *C*. *albican*s mono-cultures were inoculated with 5 μl of the same cell suspension as spots on YPD plates. *C*. *albicans* cultures were incubated for 16 hours at 30°C then 24 hours at room temperature (~23°C). Then, 5 μl of *P*. *aeruginosa* suspension, prepared from a 5 mL culture in LB grown for 16 hours then diluted in dH_2_O to OD_600_ = 2.5, was spotted on top of the *C*. *albicans* lawns for co-cultures or onto YPD for mono-cultures. Co-culture and mono-culture colony biofilms were then incubated for 16 hours at 30°C. For gradient plates (described below) 500 μl of *C*. *albicans* suspension was used to spread a lawn that was pre-grown as above and *P*. *aeruginosa* was spotted across as 12 evenly spaced 5 μl spots. All images were taken on a Canon EOS Rebel T6i camera. For visualization of siderophores, pictures were taken under UV light.

### HPLC analysis

*C*. *albicans* cultures were sub-cultured from overnight cultures grown in 5 mL YPD cultures at 30°C into fresh 5 mL YPD cultures in duplicate and incubated, aerated on a roller drum, at 30°C for 8 hours before supernatants were collected for HPLC analysis as in [26].

### RNA collection

Total RNA was harvested from *P*. *aeruginosa* mono-cultures, *C*. *albicans* mono-cultures and *P*. *aeruginosa*–*C*. *albicans* co-culture colony biofilms grown as described above. All samples were collected as cores from agar plates: cores were taken using a single-use straw, cells were suspended by shaking agar plugs in 1 mL dH_2_O on the disrupter genie for three minutes. Cells were spun down and resuspended in 1 mL Trizol and lysed by bead beating with mixed sizes of silicon beads on the Omni Bead Ruptor. Centrifugation induced phase separation and RNA was extracted from the aqueous phase where it was subsequently precipitated out with isopropanol and linear acrylamide. RNA was pelleted, washed with 70% ethanol, resuspended in nuclease-free dH_2_0 and stored at -80°C. Samples were prepared for sequencing with DNase treatment, ribodepletion and library preparation in accordance with Illumina protocols. Samples were barcoded and multiplexed in a NextSeq run for a total of 4.7x10^8^ reads.

### RNA-Seq processing

Reads were processed using the CLC Genomics Workbench with which reads were trimmed and filtered for quality using default parameters. For co-cultures, reads were first aligned to the *P*. *aeruginosa* UCBPP_PA14 genome from www.pseudomonas.com. All unaligned reads were aligned to *C*. *albicans* SC5314 genome Assembly 22 from www.candidagenome.org. For mono-cultures, reads were only aligned to their appropriate reference genome. Results were exported from CLC including total counts, CPM and TPM. R was used for principle component analysis (prcomp, stats library [104]) and consequent plotting (autoplot, ggplot2 [105]) of gene expression TPM data.

R was also used for differential gene expression analysis. EdgeR was used to process both *P*. *aeruginosa* and *C*. *albicans* gene expression separately [106]. Generalized linear models with mixed effect data design matrices were used to calculate fold-changes, p-values and FDRs for each comparison of interest. Volcano plots using EdgeR output (log_2_fold-change and -log_10_FDR) were made in R as well (ggplot2) [105].

Pathway over-representation analysis was carried out using *P*. *aeruginosa*- and *C*. *albicans*-associated KEGG pathways (ADAGEpath [64] and KEGGREST [107]) and calculated using a one-sided hypergeometric test (phyper, stats) with Bonferroni correction for multiple hypothesis testing (p.adjust, stats) [104].

### Accession number

Data for our RNA-Seq analysis of *P*. *aeruginosa* and *C*. *albicans* gene expression has been uploaded to the GEO repository (https://www.ncbi.nlm.nih.gov/geo/) with the accession number GSE148597 and raw reads are available via the SRA database with the accession number.

SRP256305 and the associated BioProject accession number PRJNA625101.

### eADAGE analysis

We performed an eADAGE analysis in accordance with the workflow published in the ADAGEpath R package [64]. Briefly, each gene expression profile in counts per million (CPM) from our RNA-Seq experiment was used to calculate lower dimensional representations of the data called signature activity profiles. Then, differentially active signatures were identified by linear model (limma, stats). This resulted in a set of signatures that were significantly different, but which may have been redundant. We applied pareto front optimization of minimal p-value and maximal absolute fold-change to arrive at a set of candidate signatures that exhibit statistically significant differences and less redundancy. Heatmaps show gene expression (CPM) or eADAGE signature activity scaled by feature (gene or signature) and are hierarchically clustered by sample using the complete method with Euclidean distance (ComplexHeatmap [108]).

### NanoString analysis

NanoString analysis was done on RNA isolated as for RNA-Seq (without DNase treatment) and 100 ng were applied to the codeset PaV5 (sequences for probesets used in this study in S4 Dataset) and processed as previously reported [2]. Counts were normalized to the geometric mean of spiked-in technical controls and five housekeeping genes (*ppiD*, *rpoD*, *soj*, *dnaN*, *pepP*, *dapF*). Normalized counts were used for heatmap construction and fold-change calculations.

### Measurement of β-galactosidase in reporter fusion strains

For co-culture promoter activity assays, *C*. *albicans* lawns were grown as described for RNA-Seq. *P*. *aeruginosa* was inoculated onto two polycarbonate filters with 0.22 μM pores (Millipore) placed on the *C*. *albicans* lawns to allow for interaction through diffusible compounds but separation of cells for quantification of promoter activity. *P*. *aeruginosa* cells were suspended in PBS by disrupter genie and diluted to OD_600_ = 0.05. β-Galactosidase (β-Gal) activity was measured as described by Miller [109]. β-Gal activity was measured in *P*. *aeruginosa* WT was normalized to that in Δ*phoB* which acted as a negative control for background-levels of PhoB-independent promoter activity.

### Assessment of phosphatase activity

For quantification of AP activity, we used a colorimetric assay using p-Nitrophenyl phosphate (pNPP) (NEB) as a substrate. Briefly, 5 μl of *P*. *aeruginosa* overnight culture was inoculated onto MOPS with 0.2% glucose and 0.7 mM phosphate agar plates on filters as for promoter activity assays with and without 1% ethanol and incubated at 37°C for 16 hours. Colony biofilms were collected from filters as described for the promoter fusion assays. 100 μl of cell suspension was added to 900 μl of 0.01 M Tris-HCl pH 8 buffer. After the addition of 25 μl 0.1% SDS and 50 μl chloroform cells were incubated at 30°C for 10 minutes. 30 μl of the aqueous phase was transferred to a 96 well plate containing 15 μl reaction buffer (5 μl 0.5 mM MgCl2 and 10 μl 1 M Tris pH 9.5) and 5 μl pNPP was added [36]. After 30 minutes OD_405_ was read on a plate reader (SpectraMax M2) and AP activity units were calculated as $\frac{{\Delta A}_{405}*ml}{10.67*min}*\frac{dilution}{A_{600}}$ where 10.67 is the extinction coefficient, normally 18.5, adjusted to the path length of the microtiter dish.

### Gradient plates

Phosphate gradient plates were made similarly to previously reported methods of creating pH gradient plates [110]. For YPD-based phosphate gradients plates, first 32 mL of molten YPD+10 mM potassium phosphate pH 6 were poured into a 10 cm square petri dish (Corning, BP124-05) that rested in a custom 3D-printed prop that held the plate slanted at a 30° angle. Once the bottom layer had solidified, the plate was laid flat and 32 mL of molten YPD agar without phosphate supplementation were poured atop. For MOPS-based gradient plates used for *P*. *aeruginosa* mono-cultures the procedure was the same except the first layer was 32 mL MOPS minimal media with 0.2% glucose and 1 mM phosphate and the top layer was 32 mL MOPS minimal medium with 0.2%glucose and 0.4 mM phosphate. When needed, BCIP was added as described above.

## Supporting information

S1 Fig Red pigment formation is dependent on phenazine biosynthesis and transport genes and quorum sensing (QS) pathways in *P*. *aeruginosa* (*P*.*a*.). A) Co- cultures of *P*.*a*. wild type (WT) and mutants lacking genes involved in phenazine biosynthesis (*phzM*, *phzS*, *phzH*) or transport (*mexGHI-ompD* and its regulator *soxR*) were inoculated onto lawns of *C*.*a*. (WT), *C*.*a*. *adh1*Δ/Δ and *adh1*Δ/Δ reconstituted with *ADH1* then incubated for 24 h. *P*.*a*. 5-MPCA phenazine production is evident by red color. B) Co-cultures of *P*.*a*. wild type (WT) and mutants lacking genes involved in quorum sensing (*lasR*, *rhlR*, *pqsR*, *pqsA*) were inoculated onto lawns of *C*.*a*. (WT), *C*.*a*. *adh1*Δ/Δ and *adh1*Δ/Δ reconstituted with *ADH1* then incubated for 48 h. C) Gene expression of *P*.*a*. genes regulated by LasR (blue), RhlR (orange) and PqsR (red) upon co-culture of *P*. *aeruginosa* WT with *C*.*a*. WT or *C*.*a*. *adh1*Δ/Δ. D) Pathway for biosynthesis in *P*. *aeruginosa* and the roles of PhzM, PhzS, and PhzH in the conversion of PCA to 5-methyl-phenazine-1-carboxylic acid (5-MPCA), pyocyanin, phenazine- 1-carboxamide (PCN) and 1-hydroxy-phenazine (1-OH-phenazine) [1].
(TIF)

S2 Fig HPLC analysis of *C*. *albicans* (*C*.*a*.*)* WT and *adh1*Δ/Δ detects differences in ethanol but not acetate or glycerol production. A) Grown in ambient oxygen, *C*.*a*. *adh1*Δ/Δ produced significantly less ethanol than WT by 4 hr and 10-fold less by 8 hr. B) At 8 hr, in atmospheric oxygen, there were no detectable differences in glycerol or acetate levels in supernatants between WT and *adh1*Δ/Δ. * p < 0.01 by ANOVA with Sidak’s multiple comparison test for both panels.
(TIF)

S3 Fig *P*. *aeruginosa* (*P*.*a*.) does not have increased colony forming units (CFU) or biomass when grown with 1% Ethanol. A) CFUs were enumerated for *P*.*a*. WT and Δ*phoB* per colony biofilm grown for 16 h from 4 μl inoculum of *P*.*a*. overnight cultures onto MOPS minimal medium agar with 0.2% glucose and 0.7 mM phosphate with and without 1% ethanol. B) OD_600_ measured for colony biofilms suspended in 1 mL dH_2_O from the conditions described in A. * p < 0.05 by two-way ANOVA with Tukey’s test for multiple comparisons.
(TIF)

S1 Table eADAGE gene-gene network cliques of DEGs from co-culture. (DOCX)

S2 Table Strains and plasmids used in this study. (DOCX)

S3 Table Primers used in this study. (DOCX)

S1 Dataset *C*.*a*. DEGS in co-culture with *P*.*a*. (XLSX)

S2 Dataset KEGG pathway analyses. (XLSX)

S3 Dataset *P*.*a*. DEGs in co-culture with *C*.*a*. (XLSX)

S4 Dataset Gene sets used throughout the paper. (XLSX)

## References

[1] Hughes WT, Kim HK. Mycoflora in cystic fibrosis: some ecologic aspects of *Pseudomonas aeruginosa* and *Candida albicans*. Mycopathol Mycol Appl. 1973;50(3):261–9. Epub 1973/07/31. 10.1007/BF02053377 . 4199669

[2] Grahl N, Dolben EL, Filkins LM, Crocker AW, Willger SD, Morrison HG, et al Profiling of bacterial and fungal microbial communities in cystic fibrosis sputum using RNA. mSphere. 2018;3(4):e00292–18. 10.1128/mSphere.00292-18 . 30089648 PMC6083091

[3] Azoulay E, Timsit J-F, Tafflet M, de Lassence A, Darmon M, Zahar J-R, et al *Candida* colonization of the respiratory tract and subsequent *Pseudomonas* ventilator-associated pneumonia. Chest. 2006;129(1):110–7. 10.1378/chest.129.1.110 16424420

[4] Falleiros de Padua RA, Norman Negri MF, Svidzinski AE, Nakamura CV, Svidzinski TI. Adherence of *Pseudomonas aeruginosa* and *Candida albicans* to urinary catheters. Rev Iberoam Micol. 2008;25(3):173–5. Epub 2008/09/13. 10.1016/s1130-1406(08)70040-8 . 18785788

[5] Gupta N, Haque A, Mukhopadhyay G, Narayan RP, Prasad R. Interactions between bacteria and *Candida* in the burn wound. Burns. 2005;31(3):375–8. 10.1016/j.burns.2004.11.012 15774298

[6] Nseir S, Jozefowicz E, Cavestri B, Sendid B, Di Pompeo C, Dewavrin F, et al Impact of antifungal treatment on *Candida*–*Pseudomonas* interaction: a preliminary retrospective case–control study. Intensive Care Med. 2007;33(1):137–42. 10.1007/s00134-006-0422-0 17115135 PMC7095372

[7] Pierce GE. *Pseudomonas aeruginosa*, *Candida albicans*, and device-related nosocomial infections: implications, trends, and potential approaches for control. J Ind Microbiol Biotechnol. 2005;32(7):309–18. 10.1007/s10295-005-0225-2 15868157

[8] Kerr JR. Suppression of fungal growth exhibited by *Pseudomonas aeruginosa*. J Clin Microbiol. 1994;32(2):525–7. 10.1128/JCM.32.2.525-527.1994 . 8150966 PMC263067

[9] Bakare N, Rickerts V, Bargon J, Just-Nubling G. Prevalence of *Aspergillus fumigatus* and other fungal species in the sputum of adult patients with cystic fibrosis. Mycoses. 2003;46(1–2):19–23. Epub 2003/02/18. 10.1046/j.1439-0507.2003.00830.x . 12588478

[10] Kaleli I, Cevahir N, Demir M, Yildirim U, Sahin R. Anticandidal activity of *Pseudomonas aeruginosa* strains isolated from clinical specimens. Mycoses. 2007;50(1):74–8. Epub 2007/02/17. 10.1111/j.1439-0507.2006.01322.x . 17302753

[11] Bauernfeind A, Bertele RM, Harms K, Horl G, Jungwirth R, Petermuller C, et al Qualitative and quantitative microbiological analysis of sputa of 102 patients with cystic fibrosis. Infection. 1987;15(4):270–7. Epub 1987/07/01. 10.1007/BF01644137 . 3117700

[12] Bandara H, Yau JYY, Watt RM, Jin LJ, Samaranayake LP. *Pseudomonas aeruginosa* inhibits *in-vitro Candida* biofilm development. BMC Microbiol. 2010;10(1):125 10.1186/1471-2180-10-125 20416106 PMC2874548

[13] Bergeron AC, Seman BG, Hammond JH, Archambault LS, Hogan DA, Wheeler RT. *Candida albicans* and *Pseudomonas aeruginosa* interact to enhance virulence of mucosal infection in transparent zebrafish. Infect Immun. 2017;85(11):e00475–17. 10.1128/IAI.00475-17 28847848 PMC5649025

[14] Brand A, Barnes JD, Mackenzie KS, Odds FC, Gow NAR. Cell wall glycans and soluble factors determine the interactions between the hyphae of *Candida albicans* and *Pseudomonas aeruginosa*. FEMS Microbiol Lett. 2008;287(1):48–55. 10.1111/j.1574-6968.2008.01301.x 18680523 PMC2613227

[15] Lopez-Medina E, Fan D, Coughlin LA, Ho EX, Lamont IL, Reimmann C, et al *Candida albicans* Inhibits *Pseudomonas aeruginosa* Virulence through Suppression of Pyochelin and Pyoverdine Biosynthesis. PLoS Path. 2015;11(8):e1005129–e. 10.1371/journal.ppat.1005129 . 26313907 PMC4552174

[16] Purschke FG, Hiller E, Trick I, Rupp S. Flexible survival strategies of *Pseudomonas aeruginosa* in biofilms result in increased fitness compared with *Candida albicans*. Molecular & Cellular Proteomics. 2012;11(12):1652–69. 10.1074/mcp.M112.017673 22942357 PMC3518115

[17] Trejo-Hernández A, Andrade-Domínguez A, Hernández M, Encarnación S. Interspecies competition triggers virulence and mutability in *Candida albicans*–*Pseudomonas aeruginosa* mixed biofilms. The ISME Journal. 2014;8(10):1974–88. 10.1038/ismej.2014.53 24739628 PMC4184018

[18] Cugini C, Morales DK, Hogan DA. *Candida albicans*-produced farnesol stimulates *Pseudomonas* quinolone signal production in LasR-defective *Pseudomonas aeruginosa* strains. Microbiology. 2010;156(Pt 10):3096–107. 10.1099/mic.0.037911-0 20656785 PMC3068698

[19] De Sordi L, Mühlschlegel FA. Quorum sensing and fungal–bacterial interactions in *Candida albicans*: a communicative network regulating microbial coexistence and virulence. FEMS Yeast Res. 2009;9(7):990–9. 10.1111/j.1567-1364.2009.00573.x 19845041

[20] Fourie R, Ells R, Swart CW, Sebolai OM, Albertyn J, Pohl CH. *Candida albicans* and *Pseudomonas aeruginosa* Interaction, with Focus on the Role of Eicosanoids. Front Physiol. 2016;7(64). 10.3389/fphys.2016.00064 26955357 PMC4767902

[21] Hogan DA, Vik Å, Kolter R. A *Pseudomonas aeruginosa* quorum-sensing molecule influences *Candida albicans* morphology. Mol Microbiol. 2004;54(5):1212–23. 10.1111/j.1365-2958.2004.04349.x 15554963

[22] Holcombe LJ, McAlester G, Munro CA, Enjalbert B, Brown AJP, Gow NAR, et al *Pseudomonas aeruginosa* secreted factors impair biofilm development in *Candida albicans*. Microbiology. 2010;156(5):1476–86. 10.1099/mic.0.037549-0. 20150241

[23] McAlester G, O'Gara F, Morrissey JP. Signal-mediated interactions between *Pseudomonas aeruginosa* and *Candida albicans*. J Med Microbiol. 2008;57(Pt 5):563–9. Epub 2008/04/26. 10.1099/jmm.0.47705-0 . 18436588

[24] Sakhtah H, Koyama L, Zhang Y, Morales DK, Fields BL, Price-Whelan A, et al The *Pseudomonas aeruginosa* efflux pump MexGHI-OpmD transports a natural phenazine that controls gene expression and biofilm development. Proc Natl Acad Sci U S A. 2016;113(25):E3538–47. 10.1073/pnas.1600424113 27274079 PMC4922186

[25] Morales DK, Jacobs NJ, Rajamani S, Krishnamurthy M, Cubillos-Ruiz JR, Hogan DA. Antifungal mechanisms by which a novel *Pseudomonas aeruginosa* phenazine toxin kills *Candida albicans* in biofilms. Mol Microbiol. 2010;78(6):1379–92. 10.1111/j.1365-2958.2010.07414.x 21143312 PMC3828654

[26] Morales DK, Grahl N, Okegbe C, Dietrich LEP, Jacobs NJ, Hogan DA. Control of *Candida albicans* metabolism and biofilm formation by *Pseudomonas aeruginosa* phenazines. mBio. 2013;4(1):e00526–12. 10.1128/mBio.00526-12 23362320 PMC3560528

[27] Gibson J, Sood A, Hogan DA. *Pseudomonas aeruginosa*-*Candida albicans* interactions: localization and fungal toxicity of a phenazine derivative. Appl Environ Microbiol. 2009;75(2):504–13. 10.1128/AEM.01037-08 19011064 PMC2620721

[28] Cugini C, Calfee MW, Farrow JM, Morales DK, Pesci EC, Hogan DA. Farnesol, a common sesquiterpene, inhibits PQS production in *Pseudomonas aeruginosa*. Mol Microbiol. 2007;65(4):896–906. 10.1111/j.1365-2958.2007.05840.x 17640272

[29] Chen AI, Dolben EF, Okegbe C, Harty CE, Golub Y, Thao S, et al *Candida albicans* ethanol stimulates *Pseudomonas aeruginosa* WspR-controlled biofilm formation as part of a cyclic relationship involving phenazines. PLoS Path. 2014;10(10):e1004480–e. 10.1371/journal.ppat.1004480 25340349 PMC4207824

[30] Kerr JR, Taylor GW, Rutman A, Høiby N, Cole PJ, Wilson R. *Pseudomonas aeruginosa* pyocyanin and 1-hydroxyphenazine inhibit fungal growth. J Clin Pathol. 1999;52(5):385–7. 10.1136/jcp.52.5.385 . 10560362 PMC1023078

[31] Harty CE, Martins D, Doing G, Mould DL, Clay ME, Occhipinti P, et al Ethanol stimulates trehalose production through a SpoT-DksA-AlgU-dependent pathway in *Pseudomonas aeruginosa*. J Bacteriol. 2019;201(12):e00794–18. 10.1128/JB.00794-18 30936375 PMC6531624

[32] Lewis KA, Baker AE, Chen AI, Harty CE, Kuchma SL, O'Toole GA, et al Ethanol decreases *Pseudomonas aeruginosa* flagellar motility through the regulation of flagellar stators. J Bacteriol. 2019;201(18):e00285–19. Epub 2019/05/22. 10.1128/JB.00285-19 . 31109994 PMC6707923

[33] DeVault JD, Kimbara K, Chakrabarty AM. Pulmonary dehydration and infection in cystic fibrosis: evidence that ethanol activates alginate gene expression and induction of mucoidy in *Pseudomonas aeruginosa*. Mol Microbiol. 1990;4(5):737–45. 10.1111/j.1365-2958.1990.tb00644.x 2167423

[34] Aendekerk S, Diggle SP, Song Z, Hoiby N, Cornelis P, Williams P, et al The MexGHI-OpmD multidrug efflux pump controls growth, antibiotic susceptibility and virulence in *Pseudomonas aeruginosa* via 4-quinolone-dependent cell-to-cell communication. Microbiology. 2005;151(Pt 4):1113–25. Epub 2005/04/09. 10.1099/mic.0.27631-0 . 15817779

[35] Bains M, Fernández L, Hancock REW. Phosphate starvation promotes swarming motility and cytotoxicity of *Pseudomonas aeruginosa*. Appl Environ Microbiol. 2012;78(18):6762–8. 10.1128/AEM.01015-12 22773629 PMC3426718

[36] Blus-Kadosh I, Zilka A, Yerushalmi G, Banin E. The effect of *pstS* and *phoB* on quorum sensing and swarming motility in *Pseudomonas aeruginosa*. PLoS One. 2013;8(9):e74444–e. 10.1371/journal.pone.0074444 24023943 PMC3762822

[37] Faure LM, Llamas MA, Bastiaansen KC, de Bentzmann S, Bigot S. Phosphate starvation relayed by PhoB activates the expression of the *Pseudomonas aeruginosa* vreI ECF factor and its target genes. Microbiology. 2013;159(Pt_7):1315–27. 10.1099/mic.0.067645-0 23657684

[38] Haddad A, Jensen V, Becker T, Haussler S. The Pho regulon influences biofilm formation and type three secretion in *Pseudomonas aeruginosa*. Environ Microbiol Rep. 2009;1(6):488–94. 10.1111/j.1758-2229.2009.00049.x 23765926

[39] Jensen V, Löns D, Zaoui C, Bredenbruch F, Meissner A, Dieterich G, et al RhlR expression in *Pseudomonas aeruginosa* is modulated by the *Pseudomonas* quinolone signal via PhoB-dependent and -independent pathways. J Bacteriol. 2006;188(24):8601–6. 10.1128/JB.01378-06 17028277 PMC1698233

[40] Lamarche MG, Wanner BL, Crépin S, Harel J. The phosphate regulon and bacterial virulence: a regulatory network connecting phosphate homeostasis and pathogenesis. FEMS Microbiol Rev. 2008;32(3):461–73. 10.1111/j.1574-6976.2008.00101.x 18248418

[41] Quesada JM, Otero-Asman JR, Bastiaansen KC, Civantos C, Llamas MA. The activity of the *Pseudomonas aeruginosa* virulence regulator σVreI is modulated by the anti-σ factor VreR and the transcription factor PhoB. Front Microbiol. 2016;7:1159-. 10.3389/fmicb.2016.01159 27536271 PMC4971064

[42] Shoriridge VD, Lazdunski A, Vasil ML. Osmoprotectants and phosphate regulate expression of phospholipase C in *Pseudomonas aeruginosa*. Mol Microbiol. 1992;6(7):863–71. 10.1111/j.1365-2958.1992.tb01537.x 1602966

[43] Zaborin A, Gerdes S, Holbrook C, Liu DC, Zaborina OY, Alverdy JC. *Pseudomonas aeruginosa* overrides the virulence inducing effect of opioids when it senses an abundance of phosphate. PLoS One. 2012;7(4):e34883–e. 10.1371/journal.pone.0034883 22514685 PMC3325935

[44] Zaborin A, Romanowski K, Gerdes S, Holbrook C, Lepine F, Long J, et al Red death in *Caenorhabditis elegans* caused by *Pseudomonas aeruginosa* PAO1. Proc Natl Acad Sci U S A. 2009;106(15):6327–32. 10.1073/pnas.0813199106 19369215 PMC2669342

[45] Chand NS, Lee JS-W, Clatworthy AE, Golas AJ, Smith RS, Hung DT. The sensor kinase KinB regulates virulence in acute *Pseudomonas aeruginosa* infection. J Bacteriol. 2011;193(12):2989–99. 10.1128/JB.01546-10 21515773 PMC3133189

[46] Cornforth DM, Dees JL, Ibberson CB, Huse HK, Mathiesen IH, Kirketerp-Møller K, et al *Pseudomonas aeruginosa* transcriptome during human infection. Proc Natl Acad Sci U S A. 2018;115(22):E5125–E34. 10.1073/pnas.1717525115 29760087 PMC5984494

[47] Cox CD, Adams P. Siderophore activity of pyoverdin for *Pseudomonas aeruginosa*. Infect Immun. 1985;48(1):130 10.1128/IAI.48.1.130-138.1985 3156815 PMC261925

[48] Damron FH, Oglesby-Sherrouse AG, Wilks A, Barbier M. Dual-seq transcriptomics reveals the battle for iron during *Pseudomonas aeruginosa* acute murine pneumonia. Sci Rep. 2016;6(1):39172-. 10.1038/srep39172 27982111 PMC5159919

[49] Damron FH, Qiu D, Yu HD. The *Pseudomonas aeruginosa* sensor kinase KinB negatively controls alginate production through AlgW-dependent MucA proteolysis. J Bacteriol. 2009;191(7):2285–95. Epub 2009/01/23. 10.1128/JB.01490-08 . 19168621 PMC2655532

[50] Francis VI, Stevenson EC, Porter SL. Two-component systems required for virulence in *Pseudomonas aeruginosa*. FEMS Microbiol Lett. 2017;364(11). 10.1093/femsle/fnx104 28510688 PMC5812489

[51] Liu PV, Shokrani F. Biological activities of pyochelins: iron-chelating agents of *Pseudomonas aeruginosa*. Infect Immun. 1978;22(3):878–90. Epub 1978/12/01. 10.1128/IAI.22.3.878-890.1978 . 103839 PMC422240

[52] Schmidberger A, Henkel M, Hausmann R, Schwartz T. Influence of ferric iron on gene expression and rhamnolipid synthesis during batch cultivation of *Pseudomonas aeruginosa* PAO1. Appl Microbiol Biotechnol. 2014;98(15):6725–37. Epub 2014/04/23. 10.1007/s00253-014-5747-y . 24752844

[53] Tan J, Doing G, Lewis KA, Price CE, Chen KM, Cady KC, et al Unsupervised extraction of stable expression signatures from public compendia with an ensemble of neural networks. Cell Systems. 2017;5(1):63–71.e6. 10.1016/j.cels.2017.06.003 28711280 PMC5532071

[54] Hogan DA, Kolter R. *Pseudomonas-Candida* interactions: an ecological role for virulence factors. Science. 2002;296(5576):2229–32. 10.1126/science.1070784 12077418

[55] Bielecki P, Jensen V, Schulze W, Gödeke J, Strehmel J, Eckweiler D, et al Cross talk between the response regulators PhoB and TctD allows for the integration of diverse environmental signals in *Pseudomonas aeruginosa*. Nucleic Acids Res. 2015;43(13):6413–25. 10.1093/nar/gkv599 26082498 PMC4513871

[56] Ching T, Himmelstein DS, Beaulieu-Jones BK, Kalinin AA, Do BT, Way GP, et al Opportunities and obstacles for deep learning in biology and medicine. Journal of The Royal Society Interface. 2018;15(141):20170387 10.1098/rsif.2017.0387 29618526 PMC5938574

[57] Greene CS, Foster JA, Stanton BA, Hogan DA, Bromberg Y. Computational approaches to study microbes and microbiomes. Pacific Symposium on Biocomputing. 2016;21:557–67. 10.1142/9789814749411_0051 26776218 PMC4832978

[58] Taroni JN, Greene CS, Martyanov V, Wood TA, Christmann RB, Farber HW, et al A novel multi-network approach reveals tissue-specific cellular modulators of fibrosis in systemic sclerosis. Genome Med. 2017;9(1):27 10.1186/s13073-017-0417-1 28330499 PMC5363043

[59] Way GP, Greene CS. Extracting a biologically relevant latent space from cancer transcriptomes with variational autoencoders. Pac Symp Biocomput. 2018;23:80–91. 10.1142/9789813235533_0008 29218871 PMC5728678

[60] Zhu Q, Wong AK, Krishnan A, Aure MR, Tadych A, Zhang R, et al Targeted exploration and analysis of large cross-platform human transcriptomic compendia. Nat Methods. 2015;12(3):211–4. 10.1038/nmeth.3249 25581801 PMC4768301

[61] Taroni JN, Grayson PC, Hu Q, Eddy S, Kretzler M, Merkel PA, et al MultiPLIER: A transfer learning framework for transcriptomics reveals systemic features of rare disease. Cell Systems. 2019;8(5):380–94.e4. Epub 2019/05/24. 10.1016/j.cels.2019.04.003 . 31121115 PMC6538307

[62] Chen KM, Tan J, Way GP, Doing G, Hogan DA, Greene CS. PathCORE-T: identifying and visualizing globally co-occurring pathways in large transcriptomic compendia. BioData Mining. 2018;11(1):14-. 10.1186/s13040-018-0175-7 29988723 PMC6029133

[63] Tan J, Hammond JH, Hogan DA, Greene CS. ADAGE-based integration of publicly svailable Pseudomonas aeruginosa gene expression data with denoising autoencoders illuminates microbe-host interactions. mSystems. 2016;1(1):e00025–15. 10.1128/mSystems.00025-15 27822512 PMC5069748

[64] Tan J, Huyck M, Hu D, Zelaya RA, Hogan DA, Greene CS. ADAGE signature analysis: differential expression analysis with data-defined gene sets. BMC Bioinformatics. 2017;18(1):512-. 10.1186/s12859-017-1905-4 29166858 PMC5700673

[65] Recinos DA, Sekedat MD, Hernandez A, Cohen TS, Sakhtah H, Prince AS, et al Redundant phenazine operons in *Pseudomonas aeruginosa* exhibit environment-dependent expression and differential roles in pathogenicity. Proceedings of the National Academy of Sciences. 2012;109(47):19420–5. 10.1073/pnas.1213901109 23129634 PMC3511076

[66] Mavrodi DV, Bonsall RF, Delaney SM, Soule MJ, Phillips G, Thomashow LS. Functional analysis of genes for biosynthesis of pyocyanin and phenazine-1-carboxamide from *Pseudomonas aeruginosa* PAO1. J Bacteriol. 2001;183(21):6454–65. 10.1128/JB.183.21.6454-6465.2001 11591691 PMC100142

[67] Lindsay AK, Morales DK, Liu Z, Grahl N, Zhang A, Willger SD, et al Analysis of *Candida albicans* mutants defective in the Cdk8 module of mediator reveal links between metabolism and biofilm formation. PLoS Genet. 2014;10(10):e1004567 Epub 2014/10/03. 10.1371/journal.pgen.1004567 . 25275466 PMC4183431

[68] Kanehisa M, Goto S. KEGG: kyoto encyclopedia of genes and genomes. Nucleic Acids Res. 2000;28(1):27–30. Epub 1999/12/11. 10.1093/nar/28.1.27 . 10592173 PMC102409

[69] Kanehisa M, Sato Y, Furumichi M, Morishima K, Tanabe M. New approach for understanding genome variations in KEGG. Nucleic Acids Res. 2019;47(D1):D590–d5. Epub 2018/10/16. 10.1093/nar/gky962 . 30321428 PMC6324070

[70] Kanehisa M. Toward understanding the origin and evolution of cellular organisms. Protein Sci. 2019;28(11):1947–51. Epub 2019/08/24. 10.1002/pro.3715 . 31441146 PMC6798127

[71] Grahl N, Demers EG, Lindsay AK, Harty CE, Willger SD, Piispanen AE, et al Mitochondrial activity and Cyr1 are key regulators of Ras1 Activation of *C*. *albicans* virulence pathways. PLoS Path. 2015;11(8):e1005133 10.1371/journal.ppat.1005133 26317337 PMC4552728

[72] Kwak MK, Ku M, Kang SO. Inducible NAD(H)-linked methylglyoxal oxidoreductase regulates cellular methylglyoxal and pyruvate through enhanced activities of alcohol dehydrogenase and methylglyoxal-oxidizing enzymes in glutathione-depleted *Candida albicans*. Biochimica et Biophysica Acta (BBA)—General Subjects. 2018;1862(1):18–39. Epub 2017/10/12. 10.1016/j.bbagen.2017.10.003 . 29017767

[73] Kwak MK, Ku M, Kang SO. NAD(+)-linked alcohol dehydrogenase 1 regulates methylglyoxal concentration in *Candida albicans*. FEBS Lett. 2014;588(7):1144–53. Epub 2014/03/13. 10.1016/j.febslet.2014.02.042 . 24607541

[74] Rampioni G, Falcone M, Heeb S, Frangipani E, Fletcher MP, Dubern JF, et al Unravelling the genome-wide contributions of specific 2-Alkyl-4-quinolones and PqsE to quorum sensing in *Pseudomonas aeruginosa*. PLoS Pathog. 2016;12(11):e1006029 Epub 2016/11/17. 10.1371/journal.ppat.1006029 . 27851827 PMC5112799

[75] Lee J, Wu J, Deng Y, Wang J, Wang C, Wang J, et al A cell-cell communication signal integrates quorum sensing and stress response. Nat Chem Biol. 2013;9(5):339–43. Epub 2013/04/02. 10.1038/nchembio.1225 . 23542643

[76] Meng X, Ahator SD, Zhang L-H. Molecular mechanisms of phosphate stress activation of *Pseudomonas aeruginosa* quorum sensing systems. mSphere. 2020;5(2):e00119–20. 10.1128/mSphere.00119-20 32188749 PMC7082139

[77] Schuster M, Lostroh CP, Ogi T, Greenberg EP. Identification, timing, and signal specificity of *Pseudomonas aeruginosa* quorum-controlled genes: a transcriptome analysis. J Bacteriol. 2003;185(7):2066–79. 10.1128/jb.185.7.2066-2079.2003 12644476 PMC151497

[78] Déziel E, Gopalan S, Tampakaki AP, Lépine F, Padfield KE, Saucier M, et al The contribution of MvfR to *Pseudomonas aeruginosa* pathogenesis and quorum sensing circuitry regulation: multiple quorum sensing-regulated genes are modulated without affecting *lasRI*, *rhlRI* or the production of N-acyl-homoserine lactones. Molecular Microbiology. 2005;55(4):998–1014. 10.1111/j.1365-2958.2004.04448.x 15686549

[79] Hammond JH, Dolben EF, Smith TJ, Bhuju S, Hogan DA. Links between Anr and Quorum Sensing in Pseudomonas aeruginosa Biofilms. J Bacteriol. 2015;197(17):2810–20. Epub 2015/06/17. 10.1128/JB.00182-15 . 26078448 PMC4524035

[80] Llamas MA, van der Sar A, Chu BCH, Sparrius M, Vogel HJ, Bitter W. A novel extracytoplasmic function (ECF) sigma factor regulates virulence in *Pseudomonas aeruginosa*. PLoS Path. 2009;5(9):e1000572 10.1371/journal.ppat.1000572 19730690 PMC2729926

[81] Filloux A, Bally M, Soscia C, Murgier M, Lazdunski A. Phosphate regulation in *Pseudomonas aeruginosa*: Cloning of the alkaline phosphatase gene and identification of *phoB*- and *phoR*-like genes. MGG Molecular & General Genetics. 1988;212(3):510–3. 10.1007/BF00330857 3138529

[82] Monds RD, Newell PD, Schwartzman JA, O'Toole GA. Conservation of the Pho regulon in *Pseudomonas fluorescens* Pf0-1. Appl Environ Microbiol. 2006;72(3):1910–24. 10.1128/AEM.72.3.1910-1924.2006 16517638 PMC1393216

[83] Monds RD, Silby MW, Mahanty HK. Expression of the Pho regulon negatively regulates biofilm formation by *Pseudomonas aureofaciens* PA147-2. Mol Microbiol. 2001;42(2):415–26. 10.1046/j.1365-2958.2001.02641.x 11703664

[84] Horwitz JP, Chua J, Noel M, Donatti JT, Freisler J. Substrates for cytochemical demonstration of enzyme activity. II. Some dihalo-3-indolyl phosphates and sulfates. Journal of Medical Chemistry. 1966;9(3):447 Epub 1966/05/01. 10.1021/jm00321a059 . 5960940

[85] Chamnongpol S, Groisman EA. Acetyl phosphate-dependent activation of a mutant PhoP response regulator that functions independently of its cognate sensor kinase. J Mol Biol. 2000;300(2):291–305. 10.1006/jmbi.2000.3848 10873466

[86] Deretic V, Leveau JHJ, Mohr CD, Hibler NS. In vitro phosphorylation of AlgR, a regulator of mucoidy in *Pseudomonas aeruginosa*, by a histidine protein kinase and effects of small phospho-donor molecules. Mol Microbiol. 1992;6(19):2761–7. 10.1111/j.1365-2958.1992.tb01455.x 1435255

[87] Hiratsu K, Nakata A, Shinagawa H, Makino K. Autophosphorylation and activation of transcriptional activator PhoB of *Escherichia coli* by acetyl phosphate in vitro. Gene. 1995;161(1):7–10. 10.1016/0378-1119(95)00259-9 7642140

[88] Kim S-K, Wilmes-Riesenberg MR, Wanner BL. Involvement of the sensor kinase EnvZ in the in vivo activation of the response-regulator PhoB by acetyl phosphate. Mol Microbiol. 1996;22(1):135–47. 10.1111/j.1365-2958.1996.tb02663.x 8899716

[89] Ikeh MAC, Kastora SL, Day AM, Herrero-de-Dios CM, Tarrant E, Waldron KJ, et al Pho4 mediates phosphate acquisition in *Candida albicans* and is vital for stress resistance and metal homeostasis. Mol Biol Cell. 2016;27(17):2784–801. 10.1091/mbc.E16-05-0266 . 27385340 PMC5007097

[90] Liu N-N, Flanagan PR, Zeng J, Jani NM, Cardenas ME, Moran GP, et al Phosphate is the third nutrient monitored by TOR in *Candida albicans* and provides a target for fungal-specific indirect TOR inhibition. Proceedings of the National Academy of Sciences. 2017;114(24):6346–51. 10.1073/pnas.1617799114 28566496 PMC5474788

[91] Lev S, Djordjevic JT. Why is a functional PHO pathway required by fungal pathogens to disseminate within a phosphate-rich host: A paradox explained by alkaline pH-simulated nutrient deprivation and expanded PHO pathway function. PLoS Path. 2018;14(6):e1007021–e. 10.1371/journal.ppat.1007021 . 29928051 PMC6013017

[92] Liu N-N, Uppuluri P, Broggi A, Besold A, Ryman K, Kambara H, et al Intersection of phosphate transport, oxidative stress and TOR signalling in *Candida albicans* virulence. PLoS Path. 2018;14(7):e1007076 10.1371/journal.ppat.1007076 30059535 PMC6085062

[93] Urrialde V, Prieto D, Pla J, Alonso-Monge R. The *Candida albicans* Pho4 transcription factor mediates susceptibility to stress and influences fitness in a mouse commensalism model. Front Microbiol. 2016;7(1062). 10.3389/fmicb.2016.01062 27458452 PMC4935684

[94] Crocker AW, Harty CE, Hammond JH, Willger SD, Salazar P, Botelho NJ, et al *Pseudomonas aeruginosa* ethanol oxidation by AdhA in low oxygen environments. J Bacteriol. 2019:JB.00393-19. 10.1128/jb.00393-19 31527114 PMC6832066

[95] Mern DS, Ha S-W, Khodaverdi V, Gliese N, Görisch H. A complex regulatory network controls aerobic ethanol oxidation in *Pseudomonas aeruginosa*: indication of four levels of sensor kinases and response regulators. Microbiology. 2010;156(5):1505–16. 10.1099/mic.0.032847-0 20093290

[96] Glasser NR, Kern SE, Newman DK. Phenazine redox cycling enhances anaerobic survival in *Pseudomonas aeruginosa* by facilitating generation of ATP and a proton-motive force. Mol Microbiol. 2014;92(2):399–412. Epub 2014/03/19. 10.1111/mmi.12566 . 24612454 PMC4046897

[97] Görisch H. The ethanol oxidation system and its regulation in *Pseudomonas aeruginosa*. Biochim Biophys Acta. 2003;1647(1):98–102. 10.1016/S1570-9639(03)00066-9. 12686116

[98] Hornby JM, Jensen EC, Lisec AD, Tasto JJ, Jahnke B, Shoemaker R, et al Quorum sensing in the dimorphic fungus *Candida albicans* is mediated by farnesol. Appl Environ Microbiol. 2001;67(7):2982–92. 10.1128/AEM.67.7.2982-2992.2001 . 11425711 PMC92970

[99] Díaz-Pérez AL, Zavala-Hernández AN, Cervantes C, Campos-García J. The *gnyRDBHAL* cluster is involved in acyclic isoprenoid degradation in *Pseudomonas aeruginosa*. Appl Environ Microbiol. 2004;70(9):5102–10. 10.1128/AEM.70.9.5102-5110.2004 . 15345388 PMC520886

[100] Bertani G. Studies on lysogenesis. I. The mode of phage liberation by lysogenic *Escherichia coli*. J Bacteriol. 1951;62(3):293–300. Epub 1951/09/01. 10.1128/JB.62.3.293-300.1951 . 14888646 PMC386127

[101] Shanks RM, Caiazza NC, Hinsa SM, Toutain CM, O'Toole GA. *Saccharomyces cerevisiae*-based molecular tool kit for manipulation of genes from gram-negative bacteria. Appl Environ Microbiol. 2006;72(7):5027–36. 10.1128/AEM.00682-06 . 16820502 PMC1489352

[102] Gibson DG, Glass JI, Lartigue C, Noskov VN, Chuang R-Y, Algire MA, et al Creation of a bacterial cell controlled by a chemically synthesized genome. Science. 2010;329(5987):52–6. 10.1126/science.1190719 20488990

[103] Gibson DG, Young L, Chuang R-Y, Venter JC, Hutchison CA, Smith HO. Enzymatic assembly of DNA molecules up to several hundred kilobases. Nat Methods. 2009;6(5):343–5. 10.1038/nmeth.1318 19363495

[104] Team RDC. R: A language and environment for statistical computing. Vienna, SAustria: R Foundation for Statistical Computing; 2010.

[105] Wickham H. ggplot2: Elegent Graphics for Data Analysis. New York: Springer-Verlag; 2016.

[106] Robinson M, McCarthy D, Smyth G. edgeR: a Bioconductor package for differential expression analysis of digital gene expression data. Bioinformatics. 2010;26(1):139–40. 10.1093/bioinformatics/btp616 19910308 PMC2796818

[107] Tenenbaum D. KEGGREST: Client-side REST access to KEGG. 2018.

[108] Zuguang G, Eils R, Schlesner M. Complex heatmaps reveal patterns and correlations in multidimensional genomic data. Bioinformatics. 2016. 10.1093/bioinformatics/btw313 27207943

[109] Miller JH. A Short Course in Bacterial Genetics: Cold Spring Harbor Press; 1992 456 p.

[110] Sacks LE. A pH gradient agar plate. Nature. 1956;178(4527):269–70. 10.1038/178269a0 13358718

