# Supplementary figures and images for: Conditional antagonism in co-cultures of Pseudomonas aeruginosa and Candida albicans: An intersection of ethanol and phosphate signaling distilled from dual-seq transcriptomics
Source: PLoS Genet. 2020 Aug 19;16(8):e1008783. doi: 10.1371/journal.pgen.1008783 (PMC7480860; doi:10.1371/journal.pgen.1008783)

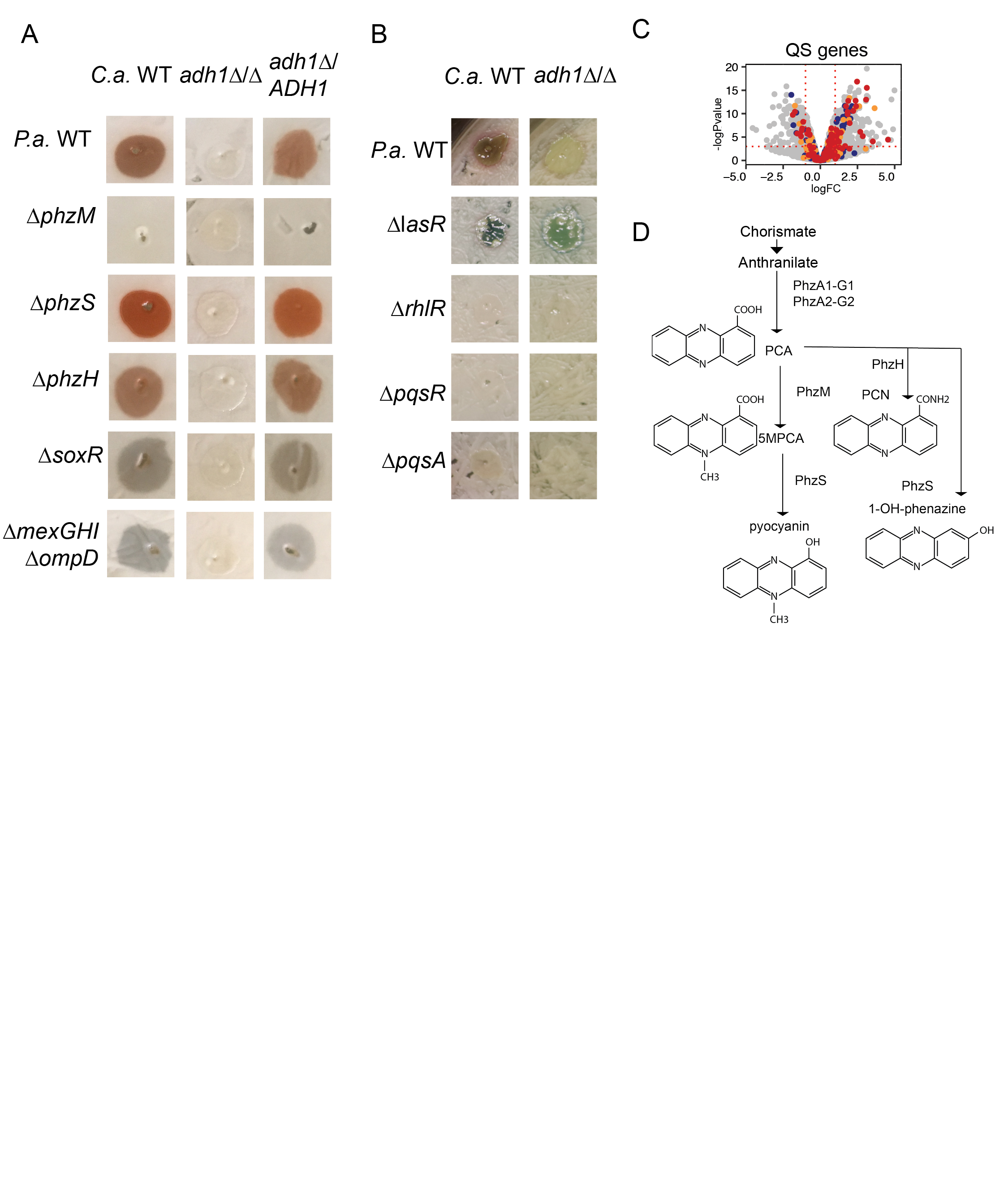

Supplement: S1 Fig — A) Co- cultures of P.a. wild type (WT) and mutants lacking genes involved in phenazine biosynthesis (phzM, phzS, phzH) or transport (mexGHI-ompD and its regulator soxR) were inoculated onto lawns of C.a. (WT), C.a. adh1Δ/Δ and adh1Δ/Δ reconstituted with ADH1 then incubated for 24 h. P.a. 5-MPCA phenazine production is evident by red color. B) Co-cultures of P.a. wild type (WT) and mutants lacking genes involved in quorum sensing (lasR, rhlR, pqsR, pqsA) were inoculated onto lawns of C.a. (WT), C.a. adh1Δ/Δ and adh1Δ/Δ reconstituted with ADH1 then incubated for 48 h. C) Gene expression of P.a. genes regulated by LasR (blue), RhlR (orange) and PqsR (red) upon co-culture of P. aeruginosa WT with C.a. WT or C.a. adh1Δ/Δ. D) Pathway for biosynthesis in P. aeruginosa and the roles of PhzM, PhzS, and PhzH in the conversion of PCA to 5-methyl-phenazine-1-carboxylic acid (5-MPCA), pyocyanin, phenazine- 1-carboxamide (PCN) and 1-hydroxy-phenazine (1-OH-phenazine) [1]. (TIF) [file pgen.1008783.s001.tif]

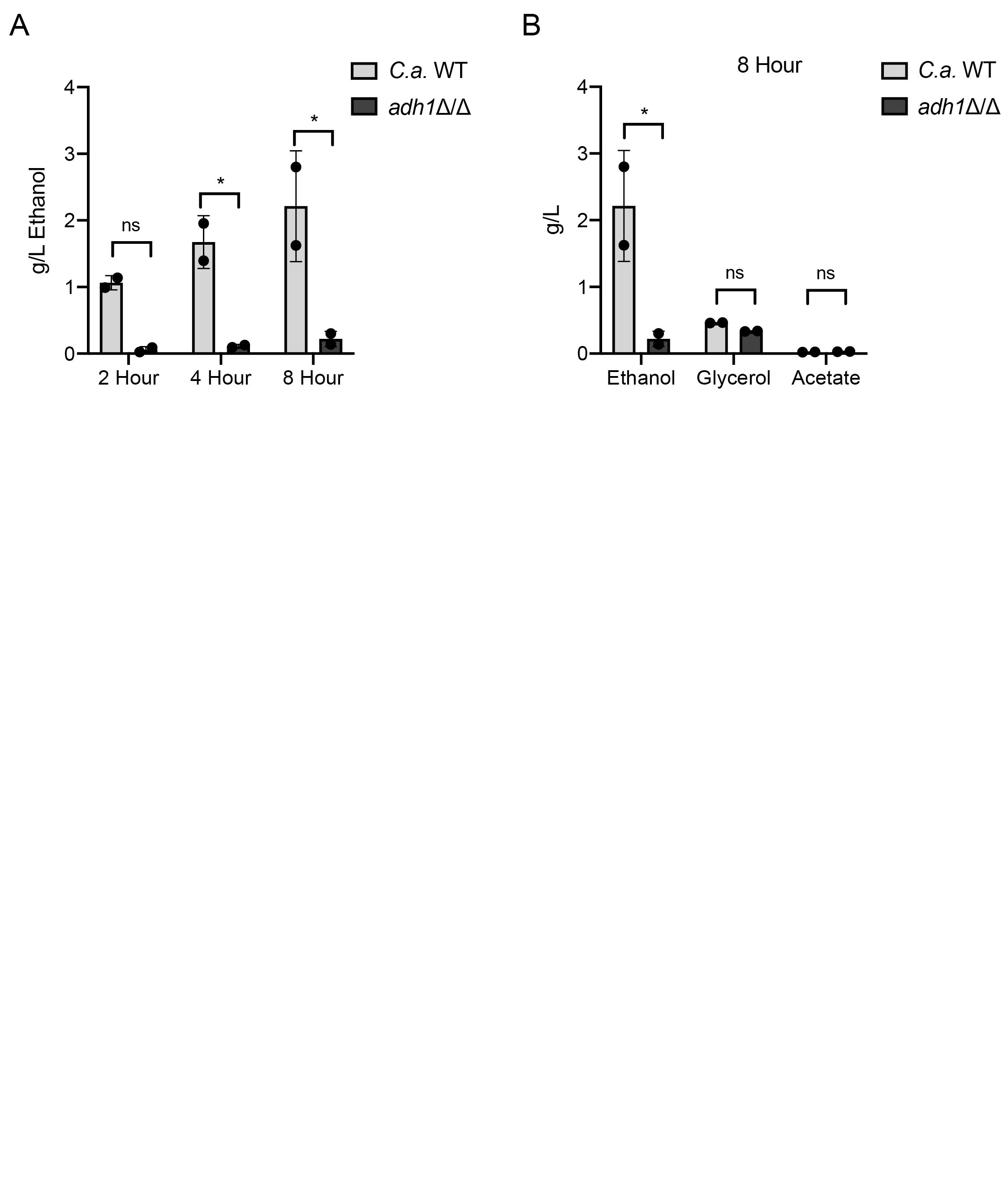

Supplement: S2 Fig — A) Grown in ambient oxygen, C.a. adh1Δ/Δ produced significantly less ethanol than WT by 4 hr and 10-fold less by 8 hr. B) At 8 hr, in atmospheric oxygen, there were no detectable differences in glycerol or acetate levels in supernatants between WT and adh1Δ/Δ. * p < 0.01 by ANOVA with Sidak’s multiple comparison test for both panels. (TIF) [file pgen.1008783.s002.tif]

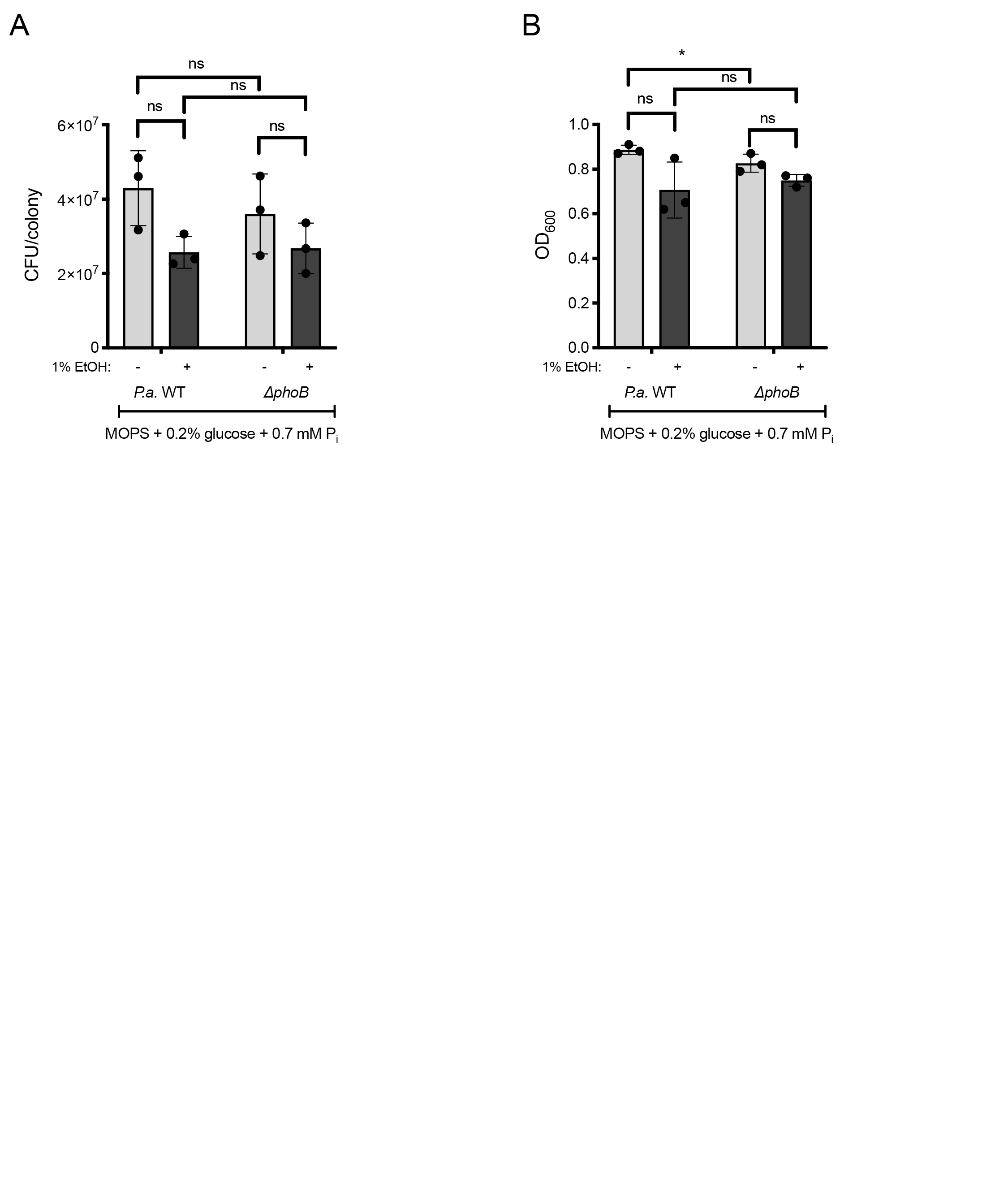

Supplement: S3 Fig — A) CFUs were enumerated for P.a. WT and ΔphoB per colony biofilm grown for 16 h from 4 μl inoculum of P.a. overnight cultures onto MOPS minimal medium agar with 0.2% glucose and 0.7 mM phosphate with and without 1% ethanol. B) OD600 measured for colony biofilms suspended in 1 mL dH2O from the conditions described in A. * p < 0.05 by two-way ANOVA with Tukey’s test for multiple comparisons. (TIF) [file pgen.1008783.s003.tif]
