# Supplementary material for: Conditional antagonism in co-cultures of Pseudomonas aeruginosa and Candida albicans: An intersection of ethanol and phosphate signaling distilled from dual-seq transcriptomics
Source: PLoS Genet. 2020 Aug 19;16(8):e1008783. doi: 10.1371/journal.pgen.1008783 (PMC7480860; doi:10.1371/journal.pgen.1008783)
Supplement: S1 Table — (DOCX) [file pgen.1008783.s004.docx]

**S1 Table. eADAGE gene-gene network cliques of DEGs from co-culture.** Cliques from the eADAGE gene-gene subnetwork of genes differentially expressed between *P.a*. grown on WT and *adh1*∆/∆ *C.a*.. Cliques were considered as groups of genes (> 1 gene) connected by edges with weights > 0.5 of Pearson correlation in signature weights in eADAGE. Cliques were numbered arbitrarily and named to represent the known functional characterizations of genes contained in each clique.

| Clique Number | Description | Clique Size | Genes |
| --- | --- | --- | --- |
| 1 | Phenazine biosynthesis | 17 | PA0522, PA3039, PA5383, PA1219, PA1221, PA1220, PA1216, PA1218, *phzA1, phzG2, phzE2, phzC2, phzD2, phzM, phzS, phzB1, phzF2* |
| 2 | Pyochelin biosynthesis | 29 | *fepD, fepB, PA4155, foxI, pvdS, femI, fiuI, femR,* PA5217, PA1301 PA1300, *hasAP*, PA4570, *pchR, fumC1, sodM*, PA4471, *fiuR, pchA, ampO, pchD, pchB, fptA, pchC, pchE,* PA4220, PA4222, PA4223, *pchF* |
| 3 | Phosphate transport and acquisition | 23 | PA2120, *hocS, phoB, pstA, pstC, plcN,* PA3383, *exbB2, phdA*, *pdtB*, PA0701, PA0699, *hxcX*, *lapA*, *exbD2*, PA0696, PA0697, PA0698, *hxcQ,* PA0700, *hxcZ*, PA0695, *pdtA* |
| 4 | Isoprenoid metabolism | 7 | *liuD, liuB, mmsB*, PA2557, PA2553, *liuC, liuA* |
| 5 | Magnesium transport | 6 | *mgtA*, PA4826, PA4635, PA4822, PA4824, PA4823 |
| 6 | Aconitate porin | 4 | *opdH*, PA0753, PA0752, PA0754 |
| 7 | Pyrimidine metabolism | 3 | *dht,* PA0440, PA0439 |
| 8 | Pyocin Biosynthesis | 3 | PA0629, PA0630, PA0637 |
| 9 | Spermidine biosynthesis | 3 | PA4773, PA4774, PA4782 |
| 10 | Histidine catabolism | 2 | *hutH*, PA5096 |
| 11 | Heat shock response | 2 | *hscA, fdx2* |
| 12 | Reactive oxygen stress response | 2 | *ahpF, katB* |
