# Supplementary material for: Conditional antagonism in co-cultures of Pseudomonas aeruginosa and Candida albicans: An intersection of ethanol and phosphate signaling distilled from dual-seq transcriptomics
Source: PLoS Genet. 2020 Aug 19;16(8):e1008783. doi: 10.1371/journal.pgen.1008783 (PMC7480860; doi:10.1371/journal.pgen.1008783)
Supplement: S2 Table — (DOCX) [file pgen.1008783.s005.docx]

**S2 Table**. **Strains and plasmids used in this study.**

| **Strain** | **Lab stock #** | **Strain description** | **Source** |
| --- | --- | --- | --- |
| ***P. aeruginosa*** |  |  |  |
| PA14 WT | DH123 | Laboratory reference strain | [1] |
| PA14 ∆*phoB* | DH3599 | Deletion mutant in *phoB* | This study |
| PA14 ∆*phoB* + *phoB* | DH3600 | Native locus complementation of *phoB* | This study |
| PA14 ∆*phoR* | DH3774 | Deletion mutant of *phoR* | [2] |
| PA14 ∆*phoB* + *phoR* | DH3755 | Plasmid-based arabinose inducible over-expression vector of *phoR* | This study |
| PA14 ∆*pstB* | DH3601 | Deletion mutant of *pstB* | This study |
| PA14 *pstB*::*Tn*M | DH753 | Transposon insertion mutant of *pstB* | [3] |
| PA14 ∆*phz* | DH933 | Deletion mutant of *phzA1-G1* and *phzA2-G2* | [4] |
| PA14 ∆*phzA1* | DH1728 | Deletion mutant of *phzA1* | [4] |
| PA14 ∆*phzA2* | DH1735 | Deletion mutant of *phzA2* | [4] |
| PA14 ∆*phzM* | DH944 | Deletion mutant of *phzM* | [5] |
| PA14 ∆*mexGHI*∆*ompD* | DH1376 | Deletion mutant of *mexGH1 and ompD* | [5] |
| PA14 ∆*soxR* | DH1377 | Deletion mutant of *soxR* | [5] |
| PA14 ∆*exaA* | DH2256 | Deletion mutant of *exaA* | [6] |
| PA14 ∆*lasR* | DH164 | Deletion mutant of *lasR* | [7] |
| PA14 ∆*rhlR* | DH2712 | Deletion mutant of *rhlR* | This study |
| PA14 ∆*psqR* | DH1110 | Deletion mutant of *pqsR* | [8] |
| PA14 ∆*pqsA* | DH556 | Deletion mutant of *pqsA* | [9] |
| PA14 ∆*exaA* + *exaA* | DH2677 | Native locus complementation of *exaA* | [6] |
| PA14 WT P*pdtA*::*lacZ*-*gfp* | DH3780 | Promoter fusion reported construct of PpdtA chromosomally integrated at the *att* site | This study |
| PA14 ∆*phoB* *PpdtA*::*lacZ*-*gfp* | DH3781 | Promoter fusion reported construct of PpdtA chromosomally integrated at the *att* site in ∆*phoB* background | This study |
| CAF2 WT | DH48 | Laboratory reference strain | [10] |
| CAF2 *adh1*∆/∆ | DH2236 | Homozygous deletion mutant of *ADH1* | [11] |
| CAF2 *adh1*∆/∆ + *ADH1* | DH2177 | Heterozygous native locus single allele complementation of *ADH1* | [11] |
| PA14 ∆*anr* | DH2855 | Deletion mutant of *anr* | [6] |
| PA14 ∆*algU* | DH3294 | Deletion mutant of *algU* | [12] |
| PA14 ∆*dksA* | DH3296 | Deletion mutant of *dksA* | [12] |
| PA14 ∆*relA* | DH3073 | Deletion mutant of *relA* | [12] |
| PA14 ∆*relA∆spoT* | DH3074 | Deletion mutant of *relA* and *spoT* | [12] |
| PA14 ∆*ackA* | DH3782 | Deletion mutant of *ackA* | [13] |
| PA14 ∆*ackA*∆*pta* | DH3783 | Deletion mutant of *ackA* and *pta* | [13] |
| PA14 ∆*ackA*∆*pta*∆*phz* | Dh3784 | Deletion mutant of *ackA, pta, phzA1-G1* and *phzA2-G2* | [13] |
| PA14 *acsA*::Tn*M* | DH2132 | ethanol catabolic transposon insertion mutant | [14] |
| PA14 Δ*kinB* | DH3778 | Deletion mutant of *kinB* | [2] |
| PA14 ∆*kinB* + *kinB* | DH3779 | Plasmid-based arabinose inducible over-expression vector of *kinB* | This study |
| PAO1 | DH3283 | Laboratory reference strain | [15] |
| PAO1 ∆*phoB* | DH3284 | Deletion mutant of *phoB* | [15] |
| PAO1 ∆*vreA* | DH3285 | Deletion mutant of *vreA* | [15] |
| PAO1 ∆*vreI* | DH3286 | Deletion mutant of *vreI* | [15] |
| PAO1 ∆*vreR* | DH3287 | Deletion mutant of *vreR* | [15] |
| PAO1 ∆*phoB*∆*vreR* | DH3288 | Deletion mutant of *phoB* and *vreR* | [15] |
| ***E. coli*** |  |  |  |
| S17 ƛpirS | DH71 | Mating competent strain used for plasmid conjugation |  |
| **Plasmids** |  |  |  |
| pMQ30 |  | allelic replacement vector, GmR | [16] |
| phoB complement |  |  | This study |
| pMQ72 |  | Arabinose inducible over-expression plasmid, empty vector | [16] |
| phoR OE |  | Arabinose inducible over-expression plasmid, containing *phoR* | This study |
| pHERD20 |  | Arabinose inducible over-expression plasmid | [17] |
| kinB OE |  | Arabinose inducible over-expression plasmid, containing *kinB* | [18] |
| P*pdtA*-*lacZ* |  | P*pdtA*-*lacZ* promoter fusion, GmR in S17 *E. coli* | This study |
| pEX18-Gm |  | Suicide vector for allelic replacement, GmR | [19] |

**References**

1. Rahme LG, Stevens EJ, Wolfort SF, Shao J, Tompkins RG, Ausubel FM. Common virulence factors for bacterial pathogenicity in plants and animals. Science. 1995;268(5219):1899-902. PubMed PMID: 7604262.

2. Tan J, Doing G, Lewis KA, Price CE, Chen KM, Cady KC, et al. Unsupervised Extraction of Stable Expression Signatures from Public Compendia with an Ensemble of Neural Networks. Cell Syst. 2017;5(1):63-71 e6. doi: 10.1016/j.cels.2017.06.003. PubMed PMID: 28711280; PubMed Central PMCID: PMCPMC5532071.

3. Liberati NT, Urbach JM, Miyata S, Lee DG, Drenkard E, Wu G, et al. An ordered, nonredundant library of *Pseudomonas aeruginosa* strain PA14 transposon insertion mutants. Proc Natl Acad Sci U S A. 2006;103(8):2833-8. Epub 2006/02/16. doi: 10.1073/pnas.0511100103. PubMed PMID: 16477005; PubMed Central PMCID: PMCPMC1413827.

4. Dietrich LEP, Price-Whelan A, Petersen A, Whiteley M, Newman DK. The phenazine pyocyanin is a terminal signalling factor in the quorum sensing network of *Pseudomonas aeruginosa*. Mol Microbiol. 2006;61(5):1308-21. doi: 10.1111/j.1365-2958.2006.05306.x.

5. Sakhtah H, Koyama L, Zhang Y, Morales DK, Fields BL, Price-Whelan A, et al. The *Pseudomonas aeruginosa* efflux pump MexGHI-OpmD transports a natural phenazine that controls gene expression and biofilm development. Proc Natl Acad Sci U S A. 2016;113(25):E3538-47. doi: 10.1073/pnas.1600424113.

6. Crocker AW, Harty CE, Hammond JH, Willger SD, Salazar P, Botelho NJ, et al. *Pseudomonas aeruginosa* ethanol oxidation by AdhA in low oxygen environments. J Bacteriol. 2019:JB.00393-19. doi: 10.1128/jb.00393-19.

7. Hogan DA, Vik Å, Kolter R. A *Pseudomonas aeruginosa* quorum-sensing molecule influences *Candida albicans* morphology. Mol Microbiol. 2004;54(5):1212-23. doi: 10.1111/j.1365-2958.2004.04349.x.

8. Cugini C, Morales DK, Hogan DA. *Candida albicans*-produced farnesol stimulates *Pseudomonas* quinolone signal production in LasR-defective *Pseudomonas aeruginosa* strains. Microbiology. 2010;156(Pt 10):3096-107. doi: 10.1099/mic.0.037911-0.

9. Deziel E, Lepine F, Milot S, He J, Mindrinos MN, Tompkins RG, et al. Analysis of *Pseudomonas aeruginosa* 4-hydroxy-2-alkylquinolines (HAQs) reveals a role for 4-hydroxy-2-heptylquinoline in cell-to-cell communication. Proc Natl Acad Sci U S A. 2004;101(5):1339-44. Epub 2004/01/24. doi: 10.1073/pnas.0307694100. PubMed PMID: 14739337; PubMed Central PMCID: PMCPMC337054.

10. Fonzi WA, Irwin MY. Isogenic strain construction and gene mapping in *Candida albicans*. Genetics. 1993;134(3):717-28. Epub 1993/07/01. PubMed PMID: 8349105; PubMed Central PMCID: PMCPMC1205510.

11. Chen AI, Dolben EF, Okegbe C, Harty CE, Golub Y, Thao S, et al. *Candida albicans* ethanol stimulates *Pseudomonas aeruginosa* WspR-controlled biofilm formation as part of a cyclic relationship involving phenazines. PLoS Path. 2014;10(10):e1004480-e. doi: 10.1371/journal.ppat.1004480.

12. Harty CE, Martins D, Doing G, Mould DL, Clay ME, Occhipinti P, et al. Ethanol stimulates trehalose production through a SpoT-DksA-AlgU-dependent pathway in *Pseudomonas aeruginosa*. J Bacteriol. 2019;201(12):e00794-18. doi: 10.1128/JB.00794-18.

13. Glasser NR, Kern SE, Newman DK. Phenazine redox cycling enhances anaerobic survival in *Pseudomonas aeruginosa* by facilitating generation of ATP and a proton-motive force. Mol Microbiol. 2014;92(2):399-412. Epub 2014/03/19. doi: 10.1111/mmi.12566. PubMed PMID: 24612454.

14. Feinbaum RL, Urbach JM, Liberati NT, Djonovic S, Adonizio A, Carvunis AR, et al. Genome-wide identification of *Pseudomonas aeruginosa* virulence-related genes using a *Caenorhabditis elegans* infection model. PLoS Pathog. 2012;8(7):e1002813. doi: 10.1371/journal.ppat.1002813. PubMed PMID: 22911607; PubMed Central PMCID: PMCPMC3406104.

15. Quesada JM, Otero-Asman JR, Bastiaansen KC, Civantos C, Llamas MA. The activity of the *Pseudomonas aeruginosa* virulence regulator σVreI is modulated by the anti-σ factor VreR and the transcription factor PhoB. Front Microbiol. 2016;7:1159-. doi: 10.3389/fmicb.2016.01159.

16. Shanks RM, Caiazza NC, Hinsa SM, Toutain CM, O'Toole GA. *Saccharomyces cerevisiae*-based molecular tool kit for manipulation of genes from gram-negative bacteria. Appl Environ Microbiol. 2006;72(7):5027-36. PubMed PMID: 16820502.

17. Qiu D, Damron FH, Mima T, Schweizer HP, Yu HD. PBAD-based shuttle vectors for functional analysis of toxic and highly regulated genes in *Pseudomonas* and *Burkholderia* spp. and other bacteria. Appl Environ Microbiol. 2008;74(23):7422-6. Epub 2008/10/14. doi: 10.1128/aem.01369-08. PubMed PMID: 18849445; PubMed Central PMCID: PMCPMC2592904.

18. Damron FH, Qiu D, Yu HD. The *Pseudomonas aeruginosa* sensor kinase KinB negatively controls alginate production through AlgW-dependent MucA proteolysis. J Bacteriol. 2009;191(7):2285-95. Epub 2009/01/23. doi: 10.1128/JB.01490-08. PubMed PMID: 19168621.

19. Hoang TT, Karkhoff-Schweizer RR, Kutchma AJ, Schweizer HP. A broad-host-range Flp-FRT recombination system for site-specific excision of chromosomally-located DNA sequences: application for isolation of unmarked *Pseudomonas aeruginosa* mutants. Gene. 1998;212(1):77-86. PubMed PMID: 9661666.
