# Supplementary material for: Conditional antagonism in co-cultures of Pseudomonas aeruginosa and Candida albicans: An intersection of ethanol and phosphate signaling distilled from dual-seq transcriptomics
Source: PLoS Genet. 2020 Aug 19;16(8):e1008783. doi: 10.1371/journal.pgen.1008783 (PMC7480860; doi:10.1371/journal.pgen.1008783)
Supplement: S3 Table — (DOCX) [file pgen.1008783.s006.docx]

**S3 Table. Primers used in this study**

|  |  |  |
| --- | --- | --- |
| **Gene/ Feature** | **Primer** | **Sequence** |
| *phoB* | phoB_KO_UP_FW | ccagggttttcccagtcacgacgttgtaaaacgacggccCCAACGCAACGACCGTCTGGC |
|  | phoB_KO_UP_RV | ccgtccaggggaaacgactccCCTCCAGGCACTCGTAGCCG |
|  | phoB_KO_DWN_FW | CGGCTACGAGTGCCTGGAGGggagtcgtttcccctggacgg |
|  | phoB_KO_DWN_RV | tgtgagcggataacaatttcacacaggaaacagctatgaccGCGGGTGTGGCGTCCAGGC |
|  | phoB_KO_CHK_FW | ccggaacctgttgagcatagccc |
|  | phoB_KO_CHK_RV | ctcctcgacatagacgttgccgc |
|  | phoB_KI_F | atacccgtttttttggg**gaaggagatatacat**ATGGTTGGCAAGACAATCCTCATCGTTG |
|  | phoB_KI_R | tctgtatcaggctgaaaatcttctctcatccgccTCAGCTCTTGGTGGAGAAACGATAGC |
|  | phoB_KI_check_F | cgtcgcacgcaccaaggcg |
|  | phoB_KI_check_R | ccctgttcgtcggcccacc |
| *pstB* | pstB_KO_UP_FW | agggttttcccagtcacgacgttgtaaaacgacggccGCGCTACAAGGTCCTGGAAGAGC |
|  | pstB_KO_UP_RV | gccgcgaccgccagagccCCGAAGACTACATCACCGGCCG |
|  | pstB_KO_Down_FW | CGGCCGGTGATGTAGTCTTCGGggctctggcggtcgcggc |
|  | pstB_KO_Down_RV | CGGCCGGTGATGTAGTCTTCGGggctctggcggtcgcggc |
|  | pstB_KO_Chk_FW | cgcctgcgcgagaagtacaagg |
|  | pstB_KO_Chk_RV | ctccacgctgaagatcgaagagctg |
| *phoR* | phoR_KI_F | catacccgtttttttggg**gaaggagatatacat**ATGCAATCCGTCGTGAACCAAGACTGG |
|  | phoR_KI_R | ttaatctgtatcaggctgaaaatcttctcTCATCCGCCTCACTTCGACGCCTTGCGCTCG |
|  | phoR_KI_check_R | gcggcaacgtctatgtcgaggag |
|  | phoR_KI_check_R | ctggtgcaggcgcagtagctgc |
| *kinB* | kinB_KI_F | atacccgtttttttggg**gaaggagatatacat**ATGGAAACCACTTCCGAAAAACAGGGGC |
|  | kinB_KI_R | atctgtatcaggctgaaaatcttctctcatccgccTCATAGGCCGTACTGCTTGCGCTTC |
|  | kinB_KI_check_R | ccgccgaatgcgcggtgacg |
|  | kinB_KI_check_R | gcggcaacgtctatgtcgaggag |
| pMQ30 | pMQ30_seq_mcs_FW | CCTCTTCGCTATTACGCCAGCTGG |
|  | pMQ30_seq_mcs_RV | GCTCACTCATTAGGCACCCCAGG |
| Reporter fusion | p pdtA 1F | GCGATTGACGGCGGGCGTCGCGATCGCCGGGGCCGCATGACTGCGGATCCCTTCCTGGAAGCTTGCCGTAC |
|  | p prhlI 2R | TTGGGACAACTCCAGTGAAAAGTTCTTCTCCTTTACTCATGACGCGAGATTCCTTGGGCGTGTTC |
| phoB sequencing | FW | cgtcgcacgcaccaaggcg |
|  | RV | ccctgttcgtcggcccacc |
| pstB sequencing | FW | ggcacggcaccaggtcagc |
|  | RV | cgacgccgcggtaggcg |
